# Mitochondria-mediated inflammation and diabetic wound healing: mechanisms and therapeutic strategies

**DOI:** 10.3389/fphar.2026.1786693

**Published:** 2026-04-01

**Authors:** Yao Chen, HuLi Li, WenJie He

**Affiliations:** 1 Pengzhou Hospital Affiliated with Chengdu University of Traditional Chinese Medicine, Pengzhou Hospital of Traditional Chinese Medicine, Pengzhou, China; 2 Department of Pharmacy, West China Hospital of Sichuan University, Chengdu, Sichuan, China; 3 Department of Pharmacy, Shenzhen Second People’s Hospital, Shenzhen, Guangdong, China

**Keywords:** diabetic wound, inflammatory response, mitochondrial dysfunction, mtDNA leakage, NLRP3 inflammasome, oxidative stress

## Abstract

Diabetic wound (DW) healing impairment is one of the most common and serious complications of diabetes. DW is characterized by a complex pathogenesis involving hyperglycemia, oxidative stress, persistent inflammation, mitochondrial dysfunction, impaired angiogenesis, and neuropathy. Recent studies have revealed that mitochondria are not only the cellular powerhouses but also key organelles regulating inflammatory responses, redox balance, and cell fate. This review summarizes how mitochondrial dysfunction exacerbates inflammation and impedes the healing process in DW through mechanisms such as excessive reactive oxygen species (ROS) production, mitochondrial DNA (mtDNA) leakage, and aberrant inflammasome activation. Furthermore, it comprehensively outlines innovative therapeutic strategies targeting mitochondria, including mitochondria-specific antioxidants, metabolic reprogramming techniques, nanomaterial-based delivery systems, genetic engineering approaches, and natural product applications. These strategies are discussed from molecular mechanisms to clinical applications, aiming to provide new insights and a theoretical basis for the clinical management of DW. Systematic analysis indicates that therapeutic strategies targeting the mitochondria-inflammation axis hold significant potential and may represent a critical breakthrough in addressing the challenge of DW healing.

## 1 Introduction

Diabetic wound (DW) is one of the most prevalent and severe complications in diabetic patients, marked by high incidence, disability, and mortality rates. Globally, approximately 15%–25% of diabetic individuals will develop a foot ulcer during their lifetime, a significant proportion of which progress to chronic, non-healing wounds, leading to amputation or death (Cano Sanchez et al., 2018; Shao et al., 2025). Statistics indicate that the risk of lower extremity amputation is 15–40 times higher in diabetic patients compared to non-diabetics, with about one million patients undergoing amputation surgeries annually due to foot complications, imposing a heavy burden on patients, families, and healthcare systems (Senneville et al., 2024). Conventional treatments such as debridement, advanced dressings, negative pressure wound therapy, and hyperbaric oxygen therapy represent the current standard of care. However, despite these interventions, clinical outcomes remain suboptimal: approximately 30%–40% of diabetic foot ulcers fail to heal within 20 weeks of standard care, and the 5-year mortality rate following amputation exceeds 70% (Chen et al., 2024; Frykberg, 2021). Even when healing is achieved, recurrence rates approach 40% within 1 year, highlighting the urgent need for more effective therapeutic strategies that target the underlying molecular mechanisms.

The impairment of DW healing is a multifactorial, multi-stage pathological process involving hyperglycemia, accumulation of advanced glycation end products (AGEs), oxidative stress, persistent inflammation, impaired angiogenesis, neuropathy, and increased infection risk (Beegum et al., 2022; He et al., 2025). These factors interact, forming a vicious cycle that prevents normal healing. Conventional treatments such as debridement, dressing changes, negative pressure wound therapy, and hyperbaric oxygen therapy can alleviate symptoms but often fail to resolve the fundamental healing impairment (Chen et al., 2024; Frykberg, 2021). Recent molecular and cell biology research has highlighted the central role of mitochondrial dysfunction—characterized by membrane potential collapse, ETC., impairment, and excessive fission—in the initiation and progression of DW (Cano Sanchez et al., 2018). Mitochondrial dysfunction encompasses a spectrum of interrelated pathological alterations in mitochondrial structure and function. First, loss of mitochondrial membrane potential (ΔΨm) represents an early and critical event, as the proton gradient across the inner mitochondrial membrane is essential for ATP synthesis through oxidative phosphorylation. When ΔΨm dissipates, the driving force for ATP production is lost, and mitochondria may undergo permeability transition, releasing pro-apoptotic factors (Peng et al., 2023) (Table 1). Second, electron transport chain (ETC.) impairment involves dysfunction of complexes I-IV, leading to reduced electron transfer efficiency, decreased ATP generation, and increased electron leakage that promotes excessive reactive oxygen species (ROS) production (Guan et al., 2022). Third, cytochrome c release from the intermembrane space into the cytosol occurs when outer mitochondrial membrane integrity is compromised, triggering apoptotic cascades through apoptosome formation and caspase activation (Li JY. et al., 2024). Fourth, mitochondrial DNA (mtDNA) damage and leakage result from its proximity to ROS production sites and lack of protective histones, making it vulnerable to oxidative modifications that can be released as damage-associated molecular patterns (DAMPs) to activate inflammatory pathways (Zhao et al., 2021). Fifth, disrupted mitochondrial dynamics refers to imbalance between fission (mediated by Drp1) and fusion (mediated by Mfn1/2 and OPA1), leading to mitochondrial fragmentation and functional decline (Chen W. et al., 2023). Finally, impaired mitophagy compromises the selective autophagic clearance of damaged mitochondria, allowing dysfunctional organelles to accumulate and perpetuate oxidative stress and inflammation (Sulkshane et al., 2021). Mitochondria are not only the primary source of ATP but also participate in regulating apoptosis, calcium homeostasis, ROS generation, and immune responses (Cano Sanchez et al., 2018; Deng et al., 2023; Qi et al., 2024) (Table 1). Under hyperglycemic conditions, dysfunction of the mitochondrial electron transport chain (ETC.) leads to excessive ROS production, subsequently inducing oxidative damage, mitochondrial DNA (mtDNA) mutations/leakage, and activation of inflammatory pathways such as the NLR family pyrin domain containing 3 (NLRP3) inflammasome and the cyclic GMP-AMP synthase-stimulator of interferon genes (cGAS-STING), thereby exacerbating local and systemic inflammation and hindering wound healing (Deng et al., 2023; Wang et al., 2025a; Öz and Çelik, 2016).

**TABLE 1:** Mitochondrial alterations in diabetic wounds: Evidence from human and animal studies.

Mitochondrial function altered	Cell type/Tissue	Species/model	Key findings	References
Membrane potential (ΔΨm) ↓	Keratinocytes, fibroblasts	Human DW tissue; db/db mice	Significant ΔΨm reduction correlates with wound severity	Deng et al. (2023), Qi et al. (2024)
mtROS production↑	Endothelial cells, macrophages	Human dermal microvascular endothelial cells; STZ-induced diabetic rats	2–3 fold increase in mtROS; precedes inflammatory activation	Dai et al. (2024), Wang et al. (2022a)
ETC., complex dysfunction (Complex I, III)	Whole wound tissue	db/db mice; human DW biopsies	Reduced complex I and III activity; increased electron leakage	Guan et al. (2022), Akude et al. (2011)
mtDNA damage and leakage	Macrophages, keratinocytes	Human DW tissue; LPS-stimulated macrophages	8-oxo-dG levels elevated; cytosolic mtDNA detected	Deng et al. (2023), He et al. (2024)
Imbalanced fission/fusion (Drp1↑, Mfn2↓)	Endothelial cells	Human dermal microvascular endothelial cells; db/db mice	Drp1 phosphorylation increased; mitochondrial fragmentation	Qi et al. (2024), Kim et al. (2018b)
Impaired mitophagy (PINK1↓, Parkin↓)	Fibroblasts, keratinocytes	Human skin fibroblasts; STZ-induced diabetic rats	Reduced autophagic flux; accumulation of damaged mitochondria	Zhang et al. (2025b), Zhang et al. (2022)
ATP production↓	Whole wound tissue	db/db mice; human DW biopsies	40%–60% reduction in ATP content vs. healthy controls	Qi et al. (2024), Ding et al. (2025)
SOD2 activity↓	Mitochondrial fraction	Human DW tissue; OLETF rats	50%–70% reduction in MnSOD activity; increased oxidative damage	Li et al. (2022a), Trambas et al. (2025)

This article systematically reviews how mitochondrial dysfunction mediates inflammatory responses to affect DW healing, providing a comprehensive analysis from molecular mechanisms and cellular levels to overall pathophysiology. It also summarizes recent advances in therapeutic strategies targeting mitochondria, including pharmacological, genetic, cellular, and material-based approaches, to offer new perspectives and directions for future treatments.

## 2 Pathophysiological basis of DW healing

### 2.1 Normal wound healing process

**FIGURE 1:**
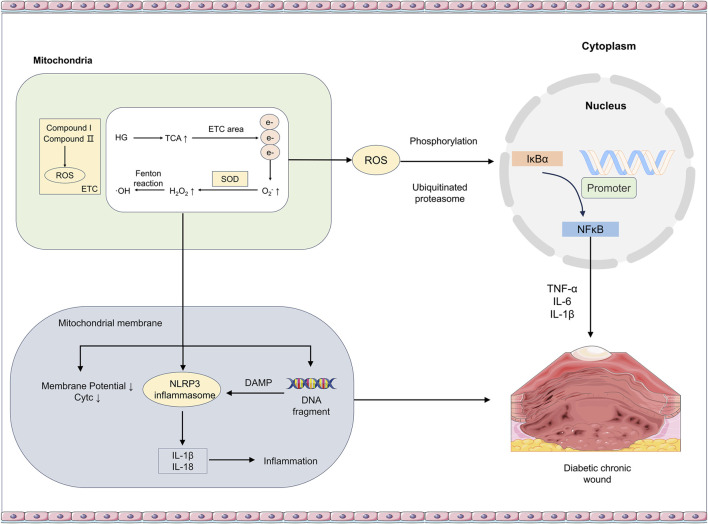
Mechanisms by which hyperglycemia (HG) triggers mitochondrial dysfunction and subsequent inflammatory responses. Hyperglycemia enhances the mitochondrial tricarboxylic acid (TCA) cycle and increases electron (e^−^) leakage from the electron transport chain (ETC.), leading to elevated reactive oxygen species (ROS) production. Concurrently, reactions such as the Fenton reaction also promote ROS generation. On one hand, ROS degrades IκBα through mechanisms like phosphorylation, allowing NF-κB dimers to translocate into the nucleus and induce the transcription of inflammatory factors such as TNF-α, IL-6, and IL-1β. On the other hand, decreased mitochondrial membrane potential, cytochrome c (Cyt c) release, and damage-associated molecular patterns (DAMPs) including DNA fragments activate the NLRP3 inflammasome. This promotes the release of IL-1β and IL-18 and, via the cGAS-STING pathway, induces pyroptosis, ultimately sustaining the inflammatory state in diabetic chronic wounds. *TCA* tricarboxylic acid, *ETC*., electron transport chain, *ROS* reactive oxygen species, *IκBα* Inhibitor of Kappa B Alpha, *NF-κB* Nuclear Factor Kappa B, *TNF-*α Tumor Necrosis Factor-Alpha, *IL-6* Interleukin-6, *IL-1*β Interleukin-1β, *Cyt c* Cytochrome c, *DAMPs* Damage-Associated Molecular Patterns, *NLRP3* NLR Family Pyrin Domain Containing 3, *IL-18* Interleukin-18, *cGAS* cyclic GMP-AMP Synthase, *STING* Stimulator of Interferon Genes.

Wound healing is a highly coordinated, dynamic process involving precise interactions among various cell types, cytokines, and extracellular matrix components. It can be divided into four overlapping yet distinct phases: hemostasis, inflammation, proliferation, and remodeling (Cano Sanchez et al., 2018; Beegum et al., 2022). The hemostatic phase begins immediately after injury, preventing further blood loss through platelet aggregation and coagulation system activation, and forming a provisional fibrin matrix that provides a scaffold for subsequent cell migration (Martin and Nunan, 2015; Renò et al., 2025). The inflammatory phase typically starts within hours post-injury and can last for several days, during which neutrophils and macrophages are recruited to the wound site to clear pathogens and necrotic tissue, while secreting various growth factors and cytokines to initiate repair (Peña and Martin, 2024; Farabi et al., 2024). The proliferation phase usually begins around days 3–4 post-injury and lasts 2–3 weeks, involving key processes such as fibroblast proliferation, collagen deposition, angiogenesis, and re-epithelialization. The remodeling phase is the longest, potentially lasting for months or years, and includes collagen reorganization and scar formation, ultimately restoring tissue integrity and function (Sideek et al., 2022; Bian et al., 2022; Guo and Dipietro, 2010). Various growth factors like PDGF, VEGF, EGF, and TGF-β play crucial roles in this process by activating specific signaling pathways that coordinate cellular activities, ensuring an orderly healing progression (Jian et al., 2022; Zhang et al., 2023; Hu et al., 2025; Ou et al., 2022) (Figure 1).

### 2.2 Pathological features of DW

Compared to normal healing, DW exhibit delayed healing and significant pathological alterations. Key characteristics include a persistent inflammatory state, oxidative stress, impaired angiogenesis, abnormal cell function, disrupted extracellular matrix (ECM) remodeling, as well as neuropathy and infection risk. In DW, the inflammatory response is abnormally prolonged, with excessive M1 macrophage polarization and sustained high expression of inflammatory cytokines (Deng et al., 2023; Monteiro et al., 2022; Mahmoud et al., 2024). Normally, inflammation should subside within days, giving way to the proliferation phase; however, in the diabetic milieu, inflammatory signaling persists, hindering the progression of healing and exacerbating oxidative stress and cellular dysfunction (Elajaili et al., 2025). Oxidative stress may initiate the inflammatory response; DW exhibit excessive ROS production and compromised antioxidant enzyme systems. Hyperglycemia causes overworking of the mitochondrial, ETC., increased electron leakage, and substantial superoxide anion generation. Concurrently, AGEs interact with their receptors (RAGE), further increasing ROS production. Oxidative stress not only directly damages biomacromolecules but also activates multiple inflammatory pathways, creating a vicious cycle (Dai et al., 2024; Wang G. et al., 2022) (Table 1).

Furthermore, the diabetic environment leads to endothelial dysfunction and insufficient angiogenesis (Wang G. et al., 2022; He et al., 2024; Ding et al., 2025). Hyperglycemia and oxidative stress cause endothelial cell dysfunction, impair VEGF signaling, reduce nitric oxide (NO) bioavailability, and diminish angiogenic capacity. Basement membrane thickening and microangiopathy further restrict blood supply and oxygen delivery, causing tissue hypoxia and impeding healing. Key repair cells such as fibroblasts, keratinocytes, and endothelial cells exhibit functional impairments, with reduced migration and proliferation capacities (Huang et al., 2023). These cells display senescent characteristics, decreased responsiveness to growth factors, and arrested cell cycle progression, leading to delayed re-epithelialization and inadequate granulation tissue formation.

Disrupted ECM remodeling is another significant factor in DW pathology. Abnormal collagen deposition and an imbalance between matrix metalloproteinases (MMPs) and their tissue inhibitors (TIMPs) occur (Shao et al., 2025; Huang et al., 2023). DW show increased MMP expression and decreased TIMP expression, resulting in excessive ECM degradation and failure to form a stable scaffold supporting cell migration and tissue reconstruction. Additionally, diabetic neuropathy causes sensory loss, potentially preventing patients from detecting and addressing wounds promptly, thereby increasing infection risk. The hyperglycemic environment provides a favorable medium for bacterial growth, and compromised immune function further elevates infection susceptibility, forming another vicious cycle that impedes healing (Bragg et al., 2024).

## 3 Mitochondrial dysfunction and inflammation in the diabetic environment

### 3.1 Mitochondrial ROS and oxidative stress

Mitochondria-derived ROS play a pivotal role in initiating the cascade of DW healing impairment. Under normal conditions, mitochondria produce ATP via oxidative phosphorylation while generating small amounts of ROS as signaling molecules. Under physiological conditions, mitochondria regulate inner membrane fluidity and ROS production through complex III of the, ETC (Liang et al., 2025; McMinimy et al., 2024). However, this balance is profoundly disrupted in a hyperglycemic environment (Cao et al., 2012; Xu et al., 2024). High glucose levels increase tricarboxylic acid cycle intermediates, delivering excess electrons to the electron transport chain (ETC.). This, ETC., overload increases electron leakage and superoxide anion generation (Gerő et al., 2016; Akude et al., 2011); these superoxide anions can be converted to hydrogen peroxide by superoxide dismutase (SOD), and further react via the Fenton reaction to produce highly reactive hydroxyl radicals, causing severe oxidative damage (Dai et al., 2024; Xu Z. et al., 2022; Thomas et al., 2009), this is the main source of mitochondrial reactive oxygen species (mtROS). Excessive ROS not only directly oxidatively damage mtDNA, leading to mutations and deletions, but also induce mitochondrial membrane lipid peroxidation and protein functional inactivation, triggering decreased mitochondrial membrane potential and cytochrome C release (Zhen et al., 2023; Mendoza et al., 2024; Wu et al., 2019; Feng et al., 2022) (Table 1).

**FIGURE 2:**
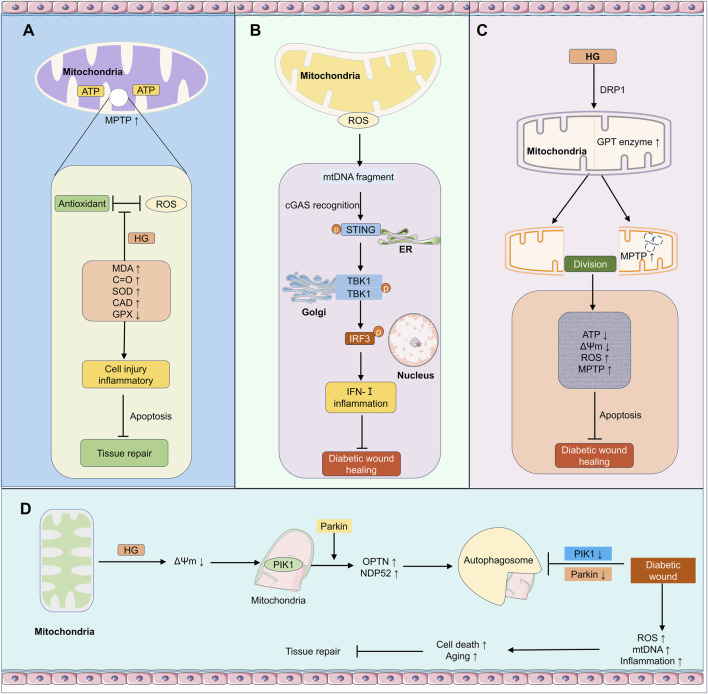
Impact of mitochondria-related mechanisms on wound healing in the diabetic environment. **(A)** Under hyperglycemia (HG), mitochondria generate ATP. While antioxidants can suppress ROS, levels of MDA, protein carbonyls (C=O), etc., increase, and changes occur in enzymes like SOD, CAT, and GPX, triggering cell damage, inflammation, and apoptosis, ultimately affecting tissue repair. **(B)** Mitochondria produce ROS, and released mitochondrial DNA (mtDNA) fragments are recognized by cGAS, activating STING and TBK1, leading to IRF3 nuclear translocation and induction of type I interferon (IFN-I)-related inflammation, hindering diabetic wound healing. **(C)** Hyperglycemia acts on mitochondria through proteins like DRP1. Involving GTPase activity and other processes, changes related to mitochondrial fission and the mitochondrial permeability transition pore (mPTP) occur, resulting in decreased ATP production, reduced membrane potential (ΔΨm), and increased ROS, leading to apoptosis and impaired diabetic wound healing. **(D)** Hyperglycemia causes a decrease in mitochondrial membrane potential. Mediated by PINK1, Parkin, and involving OPTN and NDP52, mitophagy is affected. This subsequently exacerbates inflammation through ROS, mtDNA, etc., promoting cell death and senescence, which is detrimental to diabetic wound healing. *HG* HyperGlycemia, *ATP* Adenosine Triphosphate, *ROS* reactive oxygen species, *MDA* Malondialdehyde, *SOD* Superoxide Dismutase, *CAT* Catalase, *GPX* Glutathione Peroxidase, *mtDNA* mitochondrial DNA, *cGAS* cyclic GMP-AMP Synthase, *STING* Stimulator of Interferon Genes, *TBK1* TANK-Binding Kinase 1, *IRF3* Interferon Regulatory Factor 3, *IFN-I* type I interferon, *DRP1* Dynamin-related protein 1, *GTP* Guanosine Triphosphate, *mPTP* mitochondrial Permeability Transition Pore, *ΔΨm* Mitochondrial Membrane Potential, *PINK1* PTEN-induced Kinase 1, *OPTN* Optineurin, NDP52 Nuclear dot protein 52.

The cellular antioxidant defense system, comprising both enzymatic and non-enzymatic components, normally maintains redox homeostasis. The four primary antioxidant enzymes include: superoxide dismutase (SOD), which catalyzes the dismutation of superoxide anions (O_2_^−^) to hydrogen peroxide (H_2_O_2_) and oxygen; catalase (CAT), which converts H_2_O_2_ to water and oxygen; glutathione peroxidase (GPx), which reduces H_2_O_2_ and organic hydroperoxides using reduced glutathione (GSH) as a cofactor; and glutathione reductase (GR), which regenerates GSH from oxidized glutathione (GSSG) using NADPH (Jomova et al., 2024; Djordjević et al., 2022). Of particular relevance to mitochondrial function, SOD2 (manganese-containing SOD, MnSOD) is localized exclusively in the mitochondrial matrix and represents the first line of defense against mitochondrially-derived superoxide, whereas SOD1 (copper-zinc-containing SOD, Cu/ZnSOD) is primarily cytosolic but also present in the mitochondrial intermembrane space (N and aziroğlu, 2007; Cui et al., 2024). In diabetic conditions, SOD2 expression and activity are often downregulated, compromising the mitochondrial antioxidant capacity and exacerbating mtROS accumulation (Li Q. et al., 2022; Trambas et al., 2025) (Table 1). Studies show significantly elevated ROS levels and decreased antioxidant defense system function in DW tissues. The activities of antioxidant enzymes including SOD (particularly SOD2), CAT, GPx, and GR are markedly reduced, while oxidative stress markers such as malondialdehyde (MDA) and protein carbonylation levels are significantly increased (Qian et al., 2021; Akter et al., 2025; Xie et al., 2019). This disruption of redox balance not only directly damages cellular structures and macromolecules but also serves as the fundamental trigger for activating the inflammatory pathways, ultimately exacerbating tissue damage (Wang G. et al., 2022; Tian et al., 2024; He et al., 2021; Jha et al., 2022) (Figure 2A).

### 3.2 Inflammatory pathway activation via mtROS and mtDNA leakage

#### 3.2.1 mtROS and the NLRP3 inflammasome

mtROS is a potent activator of the NLRP3 inflammasome, a multiprotein platform composed of NLRP3, ASC, and caspase-1 (Jang et al., 2015; Wang et al., 2024). Mitochondrial dysfunction plays a critical role in activating this pathway. Specifically, mtROS is believed to induce the dissociation of thioredoxin-interacting protein (TXNIP) from thioredoxin; TXNIP then directly binds to and activates the NLRP3 inflammasome (Zhou et al., 2010; Zhang et al., 2024). Recent studies suggest this process may be associated with mitochondrial iron accumulation (Ar et al., 2025). Furthermore, the mitochondrial, ETC., can also sustain NLRP3 inflammasome activation through non-ROS-dependent pathways, such as via phosphocreatine (PCr)-dependent ATP generation (Billingham et al., 2022). However, the central role of mtROS in NLRP3 activation remains contentious. Some studies have challenged this view, suggesting that inhibiting mtROS and mitochondrial function using high concentrations of chemical inhibitors can easily produce experimental artifacts (Muñoz-Planillo et al., 2013; Bauernfeind et al., 2011). These conflicting findings indicate that the role of mtROS may be influenced by experimental conditions. For instance, some research points out that under certain inhibitor treatments, ROS scavenging might concurrently interfere with the “priming signal” necessary for NLRP3 activation, thereby complicating the interpretation of results (Bauernfeind et al., 2011). Consequently, although substantial evidence supports the central importance of mtROS, its precise role and necessary conditions remain a forefront topic of intense investigation in the field. Activated caspase-1 then cleaves pro-IL-1β and pro-IL-18 into their bioactive forms, IL-1β and IL-18, which are released extracellularly, strongly amplifying local and systemic inflammatory responses (Liu et al., 2022; Ding et al., 2022). The activation level of the NLRP3 inflammasome is significantly higher in DW tissues compared to normal tissues and is closely associated with indicators of mitochondrial dysfunction (Wang et al., 2025a; He et al., 2024; Sun et al., 2023) (Figure 1).

#### 3.2.2 NF-κB pathway

Nuclear factor kappa B (NF-κB) is a master transcription factor regulating inflammatory responses (Vringer et al., 2024). In its resting state, NF-κB is bound to its inhibitory protein inhibitor of kappa b alpha (IκBα) in the cytoplasm. ROS can oxidatively modify regulatory subunits of the IκB kinase (IKK) complex, promoting IκBα phosphorylation and subsequent degradation via the ubiquitin-proteasome pathway, thereby releasing active NF-κB dimers (e.g., p50-p65) for nuclear translocation (Sá-Pessoa et al., 2023; Kim JY. et al., 2018). Within the nucleus, NF-κB binds to specific gene promoter regions, initiating the transcription of key pro-inflammatory cytokines such as tumor necrosis factor-alpha (TNF-α), IL-6, and IL-1β (Hu et al., 2020). Multiple studies confirm that in the DW microenvironment, persistent hyperglycemia-induced excessive mitochondrial ROS production leads to sustained activation of the NF-κB signaling pathway, creating a pro-inflammatory positive feedback loop that hinders the wound healing process (Wang G. et al., 2022; Tian et al., 2024) (Figure 1).

#### 3.2.3 Cytosolic mtDNA and the cGAS-STING pathway

mtDNA is particularly vulnerable to oxidative damage due to its lack of histone protection and proximity to ROS generation sites (Hayakawa et al., 1992; Barja and Herrero, 2000). The oxidative stress causes mtDNA damage and compromises mitochondrial membrane integrity, allowing mtDNA to leak into the cytosol (Wang et al., 2025a; He et al., 2024) (Table 1). This cytosolic mtDNA is recognized as a damage-associated molecular pattern (DAMP) (Zhou et al., 2011; Yu et al., 2014). It can be sensed by cyclic GMP-AMP synthase (cGAS), activating the stimulator of interferon genes (STING) pathway. Activated STING recruits TBK1, which phosphorylates IRF3, promoting the expression of type I interferons and other inflammatory cytokines (Wang et al., 2025a; He et al., 2024). It is noteworthy that in diabetic wound models using C57 mice, while the cGAS-STING pathway in macrophages becomes pathogenic due to chronic excessive activation caused by persistent mitochondrial DNA leakage (Geng et al., 2021), it may also serve as an important innate immune surveillance mechanism in skin tissue under physiological conditions. However, in some C57 mouse skin wound models, including diabetic wounds, this pathway becomes dysregulated in macrophages or endothelial cells, shifting the balance toward a chronic inflammatory state (Li F. et al., 2024; Geng et al., 2023; Chen Y. et al., 2023; Koo et al., 2019; Zhang S. et al., 2025) (Figure 2B).

### 3.3 Disruption of mitochondrial dynamics and quality control

#### 3.3.1 Imbalanced mitochondrial fission and fusion

Mitochondria are dynamic organelles that maintain network homeostasis through continuous fission and fusion (Chan, 2020; Quintana-Cabrera and Scorrano, 2023). Mitochondrial fission is mediated by Drp1, while fusion is regulated by Mfn1/2 and OPA1 (Tang et al., 2024; Kim YM. et al., 2018; Sidarala et al., 2022; Liu et al., 2021). In diabetes, this balance is disrupted, shifting towards excessive fission and resulting in mitochondrial fragmentation and functional impairment (Sun et al., 2021)**.** At the molecular level, the mechanisms governing fission and fusion are precisely regulated**.** PDIA1 binds to Drp1 to reduce its redox state. Loss of PDIA1 increases sulfenylation of Drp1 at Cys644 and enhances Drp1 activity, promoting mitochondrial fragmentation and mtROS production, which ultimately leads to endothelial cell dysfunction (Kim YM. et al., 2018) (Table 1). On the fusion side**,** Mfn1 and Mfn2 are essential for glucose-stimulated insulin secretion (GSIS) primarily by regulating mitochondrial DNA (mtDNA) content. Combined deletion of Mfn1/2 in β-cells reduces mtDNA content, impairs mitochondrial morphology and network, and compromises respiratory function, eventually resulting in severe glucose intolerance (Sidarala et al., 2022). Furthermore, CK2α-mediated Jak2-Stat3 phosphorylation activates the transcription of Opa1, thereby promoting mitochondrial fusion and suppressing mitochondrial oxidative stress (Liu et al., 2021). These mechanisms represent promising targets for overcoming mitochondrial dysfunction in diabetes and restoring glycemic control. Fragmented mitochondria are functionally deficient, producing less ATP and more ROS, and exhibit a decreased membrane potential, further promoting apoptosis (Qi et al., 2024; Sun et al., 2021). Research finds this abnormal fragmented morphology and reduced ATP output in DW tissues, failing to meet the energy demands for repair and proliferation (Deng et al., 2023; Qi et al., 2024) (Table 1). This disruption also impairs mitochondrial quality control, as fragmented mitochondria may evade autophagic clearance, accumulating and continuously generating ROS and inflammatory signals (Wang et al., 2025a) (Figure 2C).

#### 3.3.2 Mitophagy impairment

Mitophagy is a crucial quality control mechanism for selectively removing damaged mitochondria, essential for maintaining a healthy mitochondrial network (Bharath et al., 2020; Li A. et al., 2022), primarily mediated by the PINK1-Parkin pathway (Narendra and Youle, 2024; Lazarou et al., 2015; Lin et al., 2019). When mitochondrial membrane potential declines, PINK1 stabilizes on the outer mitochondrial membrane, recruiting and activating the E3 ubiquitin ligase Parkin (Table 1). Parkin then ubiquitinates outer mitochondrial membrane proteins, recruiting autophagy receptors like OPTN and NDP52, ultimately leading to the engulfment and degradation of damaged mitochondria by autophagosomes (Zhang C. et al., 2025; Wang et al., 2025b; Zhang et al., 2022) (Figure 2D).

In the diabetic environment, mitophagy is impaired, leading to the accumulation of dysfunctional mitochondria. Studies show downregulated PINK1 and Parkin expression and obstructed autophagic flux in DW tissues (Zhang C. et al., 2025). Dysfunctional mitochondria continuously produce ROS and leak mtDNA, further activating inflammatory pathways and forming a vicious cycle (Wang et al., 2025a). Mitophagy impairment also affects cell fate decisions. During wound healing, moderate autophagy is necessary for cells to adapt to stressful environments and clear damaged components. However, in diabetes, disrupted autophagy may lead to aberrant cell death or senescence, hindering tissue repair (Wang et al., 2025a; Ding et al., 2025) (Figure 2D) (Table 1).

## 4 Central role of the mitochondria-inflammation axis in DW

### 4.1 Macrophage polarization and mitochondrial metabolism

**FIGURE 3:**
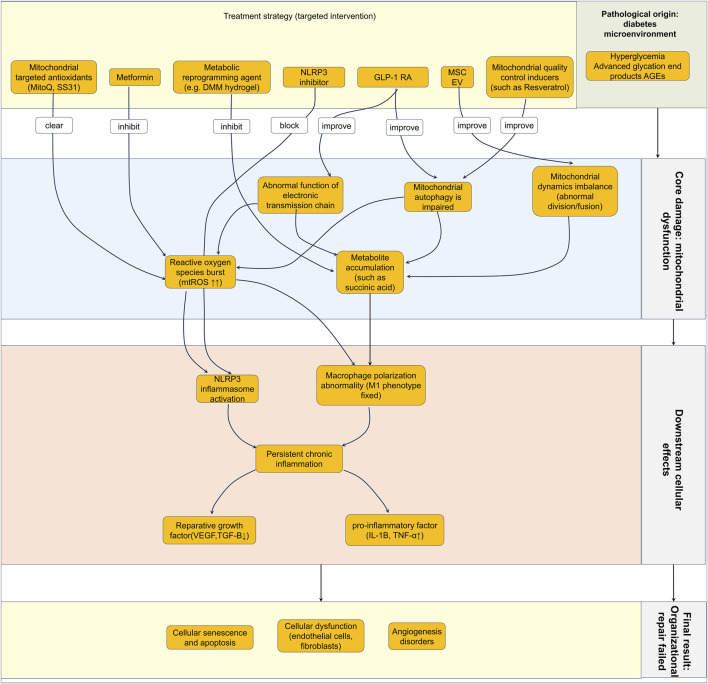
Integrated schematic of mitochondrial dysfunction as a central mechanism impairing diabetic wound healing and targeted therapeutic strategies. The figure delineates a cohesive pathological cascade from the diabetic microenvironment to failed tissue repair. The process originates from the diabetic microenvironment, characterized by hyperglycemia and accumulation of advanced glycation end products (AGEs). These factors converge to induce core damage: mitochondrial dysfunction, encompassing impaired mitochondrial dynamics, mitophagy, and electron transport chain function. This dysfunction results in metabolite accumulation (e.g., succinate) and excessive mitochondrial reactive oxygen species (mtROS) production. These primary disturbances activate two key downstream pathways: 1) NLRP3 inflammasome activation, and 2) dysregulated macrophage polarization, characterized by a sustained pro-inflammatory M1 phenotype. Together, these pathways drive a state of persistent chronic inflammation, which disrupts the balance between pro-inflammatory cytokines (e.g., IL-1β, TNF-α) and pro-repair growth factors (e.g., VEGF, TGF-β). The consequent cellular dysfunction, senescence/apoptosis, and impaired angiogenesis collectively lead to the final result: failure of tissue repair and wound healing. Abbreviations: AGEs, Advanced glycation end products; mtROS, mitochondrial reactive oxygen species; NLRP3, NLR family pyrin domain containing 3; IL-1β, Interleukin-1β; TNF-α, Tumor necrosis factor-alpha; VEGF, Vascular endothelial growth factor; TGF-β, Transforming growth factor-beta; GLP-1 RAs, Glucagon-like peptide-1 receptor agonists.

Macrophages play a dual role in wound healing, and their phenotypic polarization directly influences the healing outcome (Fu et al., 2023). Typically, macrophages are categorized into classically activated M1 (pro-inflammatory) and alternatively activated M2 (anti-inflammatory/pro-repair) types (Chen Z. et al., 2023). In normal wound healing, M1 macrophages dominate the early phase, clearing pathogens and necrotic tissue (Louiselle et al., 2021), later shifting towards the M2 phenotype to promote tissue repair and angiogenesis (Zhou et al., 2023). However, in DW, macrophage polarization is arrested in the M1 state, leading to chronic inflammation and impaired healing (Shao et al., 2025; Deng et al., 2023; Monteiro et al., 2022). Mitochondrial metabolism plays a key role in macrophage polarization. M1 macrophages primarily rely on glycolysis for energy, exhibit increased mitochondrial ROS production and succinate accumulation, promoting Hypoxia-Inducible Factor-1α (HIF-1α) stabilization and inflammatory gene expression. In contrast, M2 macrophages depend on oxidative phosphorylation (OXPHOS) and possess intact mitochondrial function and fatty acid oxidation capacity (Shao et al., 2025; Monteiro et al., 2022). Notably, the hyperglycemic and high-AGE microenvironment within the DW vasculature and surrounding tissue actively reinforces and sustains the pro-inflammatory M1 macrophage phenotype through multiple interconnected mechanisms (Fu et al., 2023). firstly, the accumulation of AGEs engages their receptor RAGE on macrophages, initiating robust pro-inflammatory signaling cascades such as the NF-κB pathway, which transcriptionally upregulates key M1 markers (e.g., TNF-α, IL-6, iNOS) while suppressing M2-associated genes like Arg1 (Dong et al., 2016; Yu et al., 2025). Secondly, hyperglycemia-driven mitochondrial dysfunction promotes the accumulation of the TCA cycle intermediate succinate, which inhibits prolyl hydroxylases (PHDs) and stabilizes HIF-1α, a master transcriptional regulator that enhances the expression of IL-1β and other M1-associated inflammatory mediators, creating a feed-forward inflammatory loop (Tannahill et al., 2013; Fuhrmann et al., 2019). Furthermore, the high-glucose environment exacerbates mtROS production, which acts as a potent signaling molecule to directly activate the NLRP3 inflammasome, thereby further cementing the M1 transcriptional program (Xu et al., 2025; Geng et al., 2024). Beyond mtROS, other danger signals including ATP, crystalline substances, and membrane damage also contribute to NLRP3 activation in the hyperglycemic, AGE-rich, and oxidative DW microenvironment (Deng et al., 2023; Wang et al., 2025a). Once activated, NLRP3 inflammasome components drive sustained IL-1β and IL-18 release, which recruit and activate neutrophils and monocytes while IL-1β promotes vascular permeability and leukocyte infiltration, and IL-18 enhances IFN-γ production—together forming an inflammatory amplification loop that perpetuates chronic inflammation and obstructs healing (Wang et al., 2025a) (Figure 3). Collectively, the synergistic action of AGE-RAGE signaling, metabolic rewiring, and oxidative stress in the DW microenvironment creates a powerful, self-reinforcing circuit that locks macrophages into a persistent M1 state, thereby perpetuating chronic inflammation and impeding the resolution of healing (Figure 3).

The diabetic environment, characterized by hyperglycemia and oxidative stress, disrupts mitochondrial function, promoting polarization towards the M1 phenotype (Luo et al., 2023; Nedosugova et al., 2022). Studies find increased expression of M1 macrophage markers and decreased expression of M2 markers in DW. Specifically, the macrophage population in DW has undergone significant changes. Compared to acute wounds, the number of M1 macrophages has increased by 2-3 fold, while M2 macrophages have decreased by approximately 50%–60%, as determined by flow cytometry and immunohistochemical analysis of wound biopsies (Deng et al., 2023; Fu et al., 2023). This polarization imbalance is reflected not only in marker expression (increased iNOS, CD86; decreased Arg1, CD206) but also in absolute cell counts, which not only maintains a chronic inflammatory state but also suppresses angiogenesis and extracellular matrix (ECM) remodeling (Shao et al., 2025; Deng et al., 2023). Modulating macrophage metabolic reprogramming has become a new therapeutic strategy. Research shows that promoting M2 polarization by upregulating arginase and downregulating inducible nitric oxide synthase, and correcting succinate dehydrogenase (SDH) in OXPHOS and the TCA cycle, can reduce inflammation (Zhao et al., 2022). Similarly, injectable hydrogels inhibiting SDH activity, reducing ROS, and promoting M2 macrophage conversion significantly improve DW healing (Shao et al., 2025). Leptin also enhances the action of IL-4 in macrophages, leading to increased oxygen consumption, upregulation of macrophage markers associated with a tissue-repair phenotype, and promotion of wound healing (Shao et al., 2025). Furthermore, a more critical perspective on the failed M2 shift reveals that it is not solely a consequence of being overwhelmed by persistent inflammatory signals. A fundamental defect in the M2-polarization program itself exists, rooted in the diabetes-induced mitochondrial damage that cripples the specific bioenergetic infrastructure required for alternative activation. Specifically, the efficient execution of fatty acid oxidation (FAO) and oxidative phosphorylation (OXPHOS)—the core metabolic pathways fueling the M2 phenotype—is severely compromised in the diabetic milieu (Purvis et al., 2024; He et al., 2019). Persistent mitochondrial dysfunction, directly undermines the cell’s capacity for OXPHOS. As demonstrated by reduced oxygen consumption rate (OCR), decreased basal and maximal respiration, and lower ATP-linked respiration in macrophages exposed to diabetic conditions (Wang L. et al., 2022; Xu L. et al., 2022). Furthermore, FAO capacity, measured by palmitate-stimulated OCR and expression of FAO enzymes (CPT1a, ACADL), is significantly impaired, preventing the metabolic reprogramming required for M2 activation (Liu et al., 2023). Consequently, even in the presence of M2-polarizing cues like IL-4, macrophages lack the functional metabolic machinery to initiate and sustain the FAO-OXPHOS metabolic program (Van den Bossche et al., 2015). This creates a dual barrier to M2 polarization: an actively hostile signaling environment that promotes M1, and a passive bioenergetic failure that prevents the cell from responding to pro-repair signals. Therefore, the failure of the M2 shift is a synergistic outcome of both sustained pro-inflammatory drivers and an intrinsic inability to utilize the specific metabolic fuels necessary for the anti-inflammatory and pro-repair functions of M2 macrophages (Figure 3).

### 4.2 Endothelial cell dysfunction

Endothelial cells play a central role in angiogenesis and maintaining vascular integrity. In diabetes, endothelial cell function is severely compromised, manifesting as decreased proliferation/migration, increased apoptosis, and impaired angiogenic capacity. Mitochondrial dysfunction plays a key role in this process (Wang G. et al., 2022; He et al., 2024; Ding et al., 2025). Fibroblasts, the primary cells responsible for ECM production and remodeling, are similarly affected by mitochondrial dysfunction in the diabetic environment. Diabetic fibroblasts exhibit mitochondrial fragmentation, reduced ATP production, and increased mtROS generation, leading to senescence and decreased proliferative capacity (Huang et al., 2023) (Zhang C. et al., 2025). This results in reduced collagen synthesis (particularly collagen type I and III) and an altered ratio of MMPs to TIMPs, favoring excessive ECM degradation (Huang et al., 2023) (Shao et al., 2025). The impaired cross-talk between dysfunctional endothelial cells and fibroblasts further compromises angiogenesis, as endothelial cells provide critical paracrine signals (such as PDGF-BB) that recruit and activate fibroblasts, while fibroblasts produce VEGF and deposit the ECM scaffold necessary for endothelial tube formation. This bidirectional dysfunction creates a self-perpetuating cycle of impaired vascularization and matrix dysregulation that severely compromises wound healing (Yang et al., 2020; Xie et al., 2013). Hyperglycemia leads to increased mitochondrial ROS production in endothelial cells, exacerbating oxidative stress. mtROS can directly damage endothelial cell DNA, proteins, and lipids, promoting senescence and apoptosis. Furthermore, mtROS can oxidize tetrahydrobiopterin (BH4), causing endothelial Nitric Oxide Synthase (eNOS) uncoupling–where eNOS produces superoxide anions instead of NO–further increasing oxidative stress and reducing NO bioavailability (Wang G. et al., 2022; Ding et al., 2025). NO is a key mediator of vasodilation and angiogenesis. Reduced NO bioavailability in diabetes leads to impaired vasodilation and angiogenesis. Concurrently, oxidative stress activates inflammatory pathways like NF-κB, promoting the expression of inflammatory cytokines and further damaging endothelial function (Wang G. et al., 2022; Ding et al., 2025) (Figure 3).

Endothelial progenitor cells (EPCs) are important for vascular repair, mobilizing from the bone marrow to the wound site to participate in neovascularization. However, diabetic patients have reduced EPC numbers and impaired function. Studies show that diabetic EPCs exhibit mitochondrial dysfunction, increased ROS production, and decreased migration and tube-forming capacity (Ding et al., 2025).

## 5 Therapeutic strategies targeting mitochondria

Given the central role of mitochondrial dysfunction in the pathogenesis of DW, developing therapeutic strategies that directly target mitochondria has become a key research focus. These approaches aim to restore mitochondrial homeostasis, correct the metabolic-inflammatory axis imbalance, and thereby promote wound healing.

### 5.1 Mitochondria-targeted antioxidants and metabolic modulators

Many natural compounds exert their therapeutic effects by improving mitochondrial function. Curcumin, at doses that achieve a hormetic effect (e.g., 10 mg/L or approximately 28 μM *in vitro*), can enhance antioxidant defense by activating the nuclear factor erythroid 2-related factor 2 (Nrf2) pathway, upregulating glutathione (GSH) metabolism, and inducing glutathione S-transferase (GST) activity, particularly isoforms involved in detoxifying lipid peroxidation products such as 4-hydroxynonenal (4-HNE); it also inhibits NF-κB-mediated inflammation (Bayet-Robert and Morvan, 2013; Piper et al., 1998) (Table 2). It is important to note that curcumin exhibits a biphasic dose-response relationship: lower doses activate protective pathways—including increased total glutathione (GSx) and GST activity—while higher doses deplete GSH, reduce related metabolites (e.g., taurine, homocysteine), and may induce pro-oxidant effects or cellular toxicity (Bayet-Robert and Morvan, 2013; Ali and Rattan, 2006). This hormetic phenomenon, where low to moderate doses stimulate adaptive protective responses and high doses become detrimental, is characteristic of many phytochemicals and must be carefully considered in therapeutic applications (Gong et al., 2023; Scharstuhl et al., 2009). Resveratrol, a natural activator of SIRT1, can deacetylate and activate key transcription factors, reducing oxidative stress and promoting mitophagy (Ciccone et al., 2022) (Table 2). These natural products function as multi-target modulators of mitochondrial health. From a genetic and cellular perspective, enhancing mitochondrial quality control is a fundamental strategy. This includes employing mitochondrial-targeted peptides and exploring gene therapies aimed at correcting defective mitophagy (e.g., via the PINK1-Parkin pathway) or normalizing imbalanced mitochondrial dynamics (Deng et al., 2023; Wang et al., 2025a; Zhang C. et al., 2025) (Tables 1, 2).

**TABLE 2:** Therapeutic strategies targeting mitochondria in diabetic wound healing.

Therapeutic strategy	Mechanism/target	Effect on wound healing	Effective model/cell type	References
MitoQ (Mitoquinone)	Mitochondria-targeted antioxidant; scavenges mtROS (Single target: mtROS)	35%–40% faster wound closure; improved re-epithelialization	Diabetic mouse wound model; Endothelial cells	Xiao et al. (2017), Zhao et al. (2019)
Elamipretide (SS-31)	Binds cardiolipin, stabilizes, ETC., reduces ROS (Single target: ETC., integrity)	Reduced wound area by 45%; decreased inflammatory markers	Diabetic mouse wound model; Macrophages	Zhao et al. (2019)
Dimethyl Malonate (DMM) hydrogel	Inhibits SDH, reduces succinate, promotes M2 polarization (Multi-target: metabolism + inflammation)	50% accelerated healing; increased M2/M1 ratio	Diabetic rat wound model; Macrophages	Shao et al. (2025), Liu et al. (2025)
CeO_2_@Tau@Hydrogel@Microneedle (CTH@MN)	Taurine-mediated anti-senescence + CeO_2_ mtROS scavenging; inhibits ROS/NF-κB pathway and activates autophagy (Multi-target: oxidative stress + inflammation + senescence)	Significantly reduced wound area; attenuated oxidative damage; decreased inflammatory cytokines; counteracted cellular senescence; promoted pro-regenerative immune microenvironment	Significantly reduced wound area; attenuated oxidative damage; decreased inflammatory cytokines; counteracted cellular senescence; promoted pro-regenerative immune microenvironment	Tian et al. (2024), Singh et al. (2023), Koo et al. (2023)
Mesoporous polydopamine NPs + SS-31	Photothermal antibacterial + mitochondrial protection (Multi-target: bacteria + mitochondria)	40% faster wound closure; enhanced re-epithelialization and collagen deposition	Diabetic mouse full-thickness wound model	Cano Sanchez et al. (2018), Deng et al. (2023)
Zn/C-dots nanozymes	SOD/CAT-like activity, scavenges ROS (Multi-target: ROS + bacteria)	Reduced wound area, bacterial load, and inflammatory cytokines	Diabetic mouse wound model	Dai et al. (2024)
Curcumin	Activates Nrf2, inhibits NF-κB (Multi-target: antioxidant + anti-inflammatory)	Improved wound healing parameters	*In vitro* endothelial cells; Diabetic rat model	Gong et al. (2023), Scharstuhl et al. (2009)
Resveratrol	Activates SIRT1, promotes mitophagy (Multi-target: autophagy + oxidative stress)	Enhanced wound closure	*In vitro* endothelial cells; Diabetic mouse model	Ciccone et al. (2022)
PINK1-Parkin pathway enhancers	Promotes mitophagy, clears damaged mitochondria (Single target: mitophagy)	35% improved wound closure; restored mitophagic flux	Diabetic mouse wound model; Keratinocytes	Zhang et al. (2025b), Wang et al. (2025b), Zhang et al. (2022)
MSC-EVs (Extracellular Vesicles)	Mitochondrial transfer, restores bioenergetics, inhibits NETs (Multi-target: energy + inflammation + angiogenesis)	Accelerated wound healing; inhibited NET-induced endothelial ferroptosis	Diabetic mouse wound model; Neutrophils, Endothelial cells	Ding et al. (2025), Lu et al. (2024)
WOC nanoplatform	Promotes M2 mitochondrial transfer to endothelial cells (Multi-target: macrophage polarization + angiogenesis)	Restored vascular function; accelerated wound healing	Diabetic mouse wound model; Macrophages, Endothelial cells	Dai et al. (2024), He et al. (2024), Qin et al. (2025), Huang et al. (2025)

Recent advances highlight several mitochondria-targeted therapeutic strategies for diabetic wound repair. The CTH@MN system (CeO_2_@Tau@Hydrogel@Microneedle) leverages taurine’s anti-senescence properties and cerium oxide’s potent mtROS-scavenging ability to inhibit the ROS/NF-κB pathway and activate autophagy (Table 2). This dual action attenuates oxidative damage and inflammation while countering cellular senescence, creating a pro-regenerative immune microenvironment that targets the “oxidation-inflammation-aging” pathological axis (Tian et al., 2024; Singh et al., 2023; Koo et al., 2023). Another emerging strategy targets mitochondrial-derived vesicles (MDVs). Under hyperglycemic conditions, upregulation of sorting nexin 9 (SNX9) triggers abnormal MDV production, transferring damaged mitochondrial components to recipient cells and impairing wound healing. Inhibiting SNX9 blocks harmful MDV formation, restores mitochondrial dynamics, and rescues healing in diabetic mice, positioning MDVs as promising therapeutic targets (Zhang H. et al., 2025). Additionally, Histatin 1 (Hst1) from human saliva accelerates wound healing by regulating mitochondria-associated endoplasmic reticulum membranes (MAMs). Hst1 inhibits the IP3R1/GRP75/VDAC1 complex, reducing MAM assembly and preventing mitochondrial calcium overload, while simultaneously inducing ERK-mediated Nrf2 nuclear translocation to enhance antioxidant defenses. This reverses endothelial cell senescence and promotes angiogenesis via the MAM-mediated mitochondria-senescence axis (Xian et al., 2024).

Conventional antioxidants are limited by poor mitochondrial specificity, but mitochondria-targeted compounds offer greater potential (Deng et al., 2023; Xiao et al., 2017). SS31 is a mitochondria-targeted peptide that specifically binds to cardiolipin, a phospholipid found exclusively on the inner mitochondrial membrane and essential for cristae formation (Chavez et al., 2020; Szeto, 2014). Through electrostatic and hydrophobic interactions with cardiolipin, SS31 prevents the oxidation of both cardiolipin and cytochrome C while disrupting their interaction (Szeto and Birk, 2014; Sweetwyne et al., 2017; Liu et al., 2019). This stabilizes the electron transport chain, enhancing electron transfer and oxidative phosphorylation, thereby reducing mitochondrial reactive oxygen species (mtROS) production and boosting ATP synthesis (Sweetwyne et al., 2017; Birk et al., 2013). The consequent decrease in mtROS and increase in ATP availability promote the polarization of macrophages toward the anti-inflammatory M2 phenotype. This shift upregulates the secretion of growth factors such as vascular endothelial growth factor (VEGF) and epidermal growth factor (EGF), ultimately enhancing cellular adhesion, proliferation, and migration (Burnstock et al., 2012; Li et al., 2019). MitoQ primarily localizes to the mitochondrial matrix, where it efficiently scavenges mtROS. Meanwhile, elamipretide acts by binding to cardiolipin, stabilizing the, ETC., supercomplexes, and reducing electron leakage, thereby decreasing mtROS production at its source (Deng et al., 2023; Zhao et al., 2019) (Table 2). While MitoQ has been shown to accumulate in mitochondrial membranes and prevent ROS accumulation in multiple tissues *in vivo*, its effects can be tissue-specific. For instance, studies indicate that mice fed a high-fat diet and treated with MitoQ exhibit minimal metabolic benefits in adipose tissue itself, despite significant improvements in systemic metabolism (Bond et al., 2019). Beyond direct antioxidant strategies, metabolic reprogramming offers a promising approach to address the underlying energy crisis in diabetic wounds (DW). For example, inhibiting succinate dehydrogenase (SDH) activity with dimethyl malonate (DMM) delivered vi*a* injectable hydrogels has been shown to reduce succinate accumulation (Table 2). This alleviates HIF-1α-mediated inflammation and promotes the repolarization of macrophages toward the M2 phenotype (Shao et al., 2025), demonstrating that correcting specific mitochondrial metabolic pathways is a viable therapeutic strategy (Liu et al., 2025) (Table 2)**.** Moreover, inhibiting NLRP3 inflammasome activation has become an important therapeutic strategy. Studies show that using NLRP3 inhibitors or interventions targeting upstream activation signals can significantly reduce inflammation and improve wound healing. For instance, mitochondria-targeted antioxidants can reduce mtROS production, indirectly suppressing NLRP3 inflammasome activation (Deng et al., 2023) (Figure 3). Research indicates that antioxidant therapy, metabolic modulation, or enhanced mitophagy can improve endothelial cell function and promote angiogenesis. For example, GLP-1 RAs can restore EPC function by improving mitochondrial function and autophagic flux; Restoring endothelial cell mitochondrial function is a crucial strategy for improving angiogenesis in DW (Lu et al., 2024). metformin can protect endothelial cells from methylglyoxal (MGO)-induced apo ptosis by inhibiting ROS (Wang G. et al., 2022; Ding et al., 2025) (Figure 3).

### 5.2 Nanomaterial-based delivery systems and mitochondrial therapy

Nanomaterials offer a powerful platform for precise mitochondrial-targeted therapy. For example, mesoporous polydopamine nanoparticles can be loaded with the mitochondria-protective peptide SS-31 and incorporated into hydrogels, achieving synergistic photothermal antibacterial effects and mitochondrial function maintenance in a diabetic mouse full-thickness wound model, resulting in 40% faster wound closure compared to controls and improved histological outcomes including enhanced re-epithelialization and collagen deposition (Deng et al., 2023) (Table 2). Carbon dot-based nanozymes (e.g., Zn/C-dots) possessing SOD and CAT-like multi-enzyme activities effectively scavenged ROS and exerted antibacterial effects against both *S. aureus* and *E. coli*. When encapsulated in ROS-responsive hydrogels, they enabled localized and sustained release tailored to the wound microenvironment, significantly reducing wound area, bacterial load, and inflammatory cytokine levels in diabetic mice (Dai et al., 2024) (Table 2). The CTH@MN system (CeO_2_@Tau@Hydrogel@Microneedle), leveraging taurine’s anti-senescence properties and cerium oxide’s mtROS-scavenging ability, successfully inhibited the ROS/NF-κB pathway and activated autophagy in diabetic wounds, attenuating oxidative damage and inflammation while countering cellular senescence to create a pro-regenerative immune microenvironment (Tian et al., 2024; Singh et al., 2023; Koo et al., 2023) (Table 2). Collectively, these nanomaterial-based approaches demonstrate that precise mitochondrial targeting, combined with microenvironment-responsive release and multi-functional activities (antioxidant, antibacterial, anti-senescence), can significantly enhance diabetic wound healing by addressing multiple pathological factors simultaneously.

### 5.3 Natural products, extracellular vesicles, and mitochondrial quality control

Emerging evidence highlights ROMO1 (Reactive Oxygen Species Modulator 1) as a critical mitochondrial inner membrane protein that governs mtROS generation and macrophage polarization in diabetic wounds. ROMO1 is known to regulate mitochondrial ROS bursts through its association with the electron transport chain, and its dysregulation has been implicated in various oxidative stress-related pathologies (Zhu et al., 2019). In the context of diabetic wound healing, a recent study developed a redox-responsive chitosan hydrogel loaded with mitochondria-targeted fullerenol (C60@QM-HG) that modulates mitochondrial redox status via the HSPA8-ROMO1 signaling axis (Wu et al., 2026). This system achieved controlled release of C60(OH)n through reversible borate ester bonds, enabling tailored antioxidant effects under diabetic wound conditions. Mechanistically, C60@QM-HG targeted mitochondria to regulate the M1/M2 macrophage balance through the HSPA8-ROMO1 pathway, resulting in significant antioxidant and anti-inflammatory effects. By suppressing M1 polarization and promoting the transition to the anti-inflammatory M2 phenotype, this intervention improved the inflammatory microenvironment, accelerated re-epithelialization and collagen deposition, and enhanced healing of diabetic and infected wounds. Furthermore, to enhance mitochondrial targeting, C60(OH)n was conjugated with Apoptozole (an HSPA8 inhibitor), yielding AP&C60@QM-HG, which further suppressed M1 polarization and promoted diabetic wound healing (Wu et al., 2026).

Mitochondrial transfer is emerging as a key therapeutic mechanism, facilitated by both engineered nanomaterials and natural vesicles. For instance, a smart tungstate-chitosan oligosaccharide (WOC) nanoplatform promotes M2 macrophage polarization and subsequent vesicle-dependent mitochondrial transfer to endothelial cells, restoring vascular function and accelerating wound healing (Dai et al., 2024; He et al., 2024; Qin et al., 2025; Huang et al., 2025) (Table 2). In parallel, mesenchymal stem cell-derived extracellular vesicles (MSC-EVs) deliver functional mitochondria to dysfunctional neutrophils in the wound site. This transfer restores neutrophil bioenergetics via mitochondrial fusion, thereby inhibiting the formation of neutrophil extracellular traps (NETs) and breaking the cycle of NET-induced endothelial ferroptosis, which ultimately promotes angiogenesis and repair (Lu et al., 2024). This multifaceted approach, often combined with anti-inflammatory signaling, positions MSC-EVs as a promising cell-free therapy (Ding et al., 2025; Lu et al., 2024) (Table 2). In addition, restoring mitophagy has emerged as a novel strategy for DW treatment. Research indicates that enhancing mitophagy through pharmacological or genetic means can significantly improve DW healing. For example, Glucagon-like peptide-1 (GLP-1) receptor agonists can restore endothelial progenitor cell function by improving mitophagic flux; natural compounds like resveratrol can promote mitophagy via Sirtuin 1 (SIRT1) activation (Ding et al., 2025) (Figure 2D).

Collectively, these mitochondria-targeted therapeutic strategies—ranging from direct antioxidant approaches and metabolic modulation to quality control enhancement and mitochondrial transfer—address distinct but interconnected aspects of mitochondrial dysfunction in diabetic wounds (Table 2). While the majority of these approaches have been validated primarily in preclinical animal models, several have established safety profiles in human trials for other indications: MitoQ has completed Phase II trials for Parkinson’s disease and hepatitis C with favorable safety outcomes (Zhang H. et al., 2025); resveratrol has been extensively studied in human metabolic disease trials (Erol Doğan et al., 2024; Corbi et al., 2023); and mesenchymal stem cell-derived extracellular vesicles are currently in early-phase clinical trials for various inflammatory conditions (Lightner et al., 2023; Chu et al., 2022). These safety data, combined with promising preclinical efficacy in DW models, support the translational potential of mitochondria-targeted interventions for diabetic wound care.

## 6 Discussion and outlook

Translating intervention strategies targeting the mitochondria-inflammation axis from basic research to clinical applications for DW still faces a series of critical challenges. First, the precision of mechanistic understanding and the complexity of translation represent the primary obstacles. Although the central roles of mtROS, mtDNA leakage, and the NLRP3/cGAS-STING pathways in driving chronic inflammation in DW have been established, the specific regulatory networks of these events across different stages of wound healing and various cell types (such as macrophages, fibroblasts, and keratinocytes) remain unclear. This directly limits the development of therapies capable of precisely interrupting the vicious cycle of inflammation without compromising normal immune surveillance.

In the advancement of treatment strategies, although approaches like mitochondria-targeted antioxidants (e.g., MitoQ) and macrophage metabolic reprogramming (e.g., SDH inhibitors) have shown significant efficacy in animal models, their clinical translation faces severe challenges in targeted delivery and microenvironment adaptation. The unique pathological microenvironment of DW—including persistently high ROS levels, high protease activity, and pH fluctuations—poses significant challenges to drug stability, retention, and bioactivity. Emerging smart nanotechnologies, such as ROS-responsive hydrogels, triggerable nanozymes (e.g., Zn/C-dots), and active mitochondrial delivery systems based on extracellular vesicles, offer breakthrough potential for achieving spatiotemporally precise drug control and release, along with synergistic antioxidant, anti-inflammatory, and antibacterial effects. However, the inherent biosafety, large-scale production processes, and cost-effectiveness of these complex systems are practical issues that must be resolved before they can enter clinical practice.

Furthermore, mitochondrial transfer, as a cutting-edge technology, provides a novel paradigm for addressing the aforementioned challenges. Research indicates that EVs derived from mesenchymal stem cells (MSCs) or M2 macrophages can horizontally transfer intact functional mitochondria to damaged cells (such as endothelial cells and inflammatory neutrophils) at the DW site. This natural “organelle replacement therapy” directly supplies healthy mitochondria to functionally compromised cells, thereby restoring cellular ATP generation capacity at the root, reducing mtROS levels, and decreasing the release of pro-inflammatory factors (e.g., IL-1β, IL-18). More intriguingly, this transfer is not merely a simple “energy transfusion”; it can also reshape the metabolic state of recipient cells, for instance, by promoting a return to OXPHOS, which is crucial for inducing macrophage polarization toward the reparative M2 phenotype. However, the translational application of this technology still requires optimization of its engineering strategies, such as improving mitochondrial loading efficiency, endowing EVs with “homing” capabilities to target specific diseased cells, and ensuring standardized and safe large-scale production.

The need for personalized medicine is particularly prominent in the field of DW. Future research must integrate multi-omics analyses to identify biomarkers that can predict therapeutic responses, thereby determining which patients are more likely to benefit from different strategies such as mitochondrial transfer, antioxidant, or anti-inflammatory therapies. The daunting task of clinical validation cannot be overlooked. Whether for EV-based biologics or complex nanomedicines, advancing their clinical application necessitates rigorously designed clinical trials to verify their safety and efficacy in promoting healing in humans.

Finally, with the global prevalence of diabetes continuing to rise, developing innovative therapies that can reverse chronic inflammation in DW and fundamentally promote healing holds significant social and economic importance. Cutting-edge technologies represented by extracellular vesicle-mediated mitochondrial transfer signify a shift in diabetic wound treatment from traditional “pharmacological intervention” to an innovative “organelle repair” era. Future research should focus on bridging the entire chain from “mechanistic understanding” to “technological innovation” and finally to “clinical validation,” employing multidisciplinary and synergistic strategies to ultimately bring truly effective mitochondria-targeted therapeutic options to DW patients.

## Glossary

AGEs: Advanced Glycation End products
AMPK: AMP-activated protein kinase
Arg1: Arginase 1
ASC: Apoptosis-associated speck-like protein containing a CARD
ATP: Adenosine Triphosphate
BA: Baicalin
BH4: Tetrahydrobiopterin
CAT: Catalase
C-dots: Carbon dots
cGAS: cyclic GMP-AMP Synthase
Cyt c: Cytochrome c
DAMPs: Damage-Associated Molecular Patterns
DW: Diabetic wounds
DMM: Dimethyl Malonate
Drp1: Dynamin-related protein 1
ECM: Extracellular Matrix
EGF: Epidermal Growth Factor
EPCs: Endothelial Progenitor Cells
ETC: Electron Transport Chain EVs Extracellular Vesicles
Fenton reaction: Fenton reaction
GPx: Glutathione Peroxidase
GTP: Guanosine Triphosphate
HG: HyperGlycemia
HIF-1α: Hypoxia-Inducible Factor 1-alpha
IFN: Interferon
IκBα: Inhibitor of Kappa B Alpha
IL: Interleukin
iNOS: inducible Nitric Oxide Synthase
IRF3: Interferon Regulatory Factor 3
MAPK: Mitogen-Activated Protein Kinase
MDA: Malondialdehyde
Mfn1/2: Mitofusin 1/2
MMPs: Matrix Metalloproteinases
MOFs: Metal-Organic Frameworks
MPs: Mitochondria-targeted Peptides
mPTP: mitochondrial Permeability Transition Pore
MSCs: Mesenchymal Stem Cells
mtDNA: mitochondrial DNA
mtROS: mitochondrial Reactive Oxygen Species
NF-κB: Nuclear Factor Kappa B
NLRP3: NLR Family Pyrin Domain Containing 3
NO: Nitric Oxide
Nrf2: Nuclear factor erythroid 2-related factor 2
OPA1: Optic Atrophy 1
OXPHOS: OXidative PHOSphorylation
PDGF: Platelet-Derived Growth Factor
PHD: Prolyl Hydroxylase
PINK1: PTEN-induced Kinase 1
RAGE: Receptor for AGEs
ROS: Reactive Oxygen Species
SDH: Succinate Dehydrogenase
SIRT1: Sirtuin 1
SOD: Superoxide Dismutase
STING: Stimulator of Interferon Genes
TCA: Tricarboxylic Acid
TGF-β: Transforming Growth Factor-Beta
TIMPs: Tissue Inhibitors of Metalloproteinases
TNF-α: Tumor Necrosis Factor-Alpha
TPP+: Triphenylphosphonium
VEGF: Vascular Endothelial Growth Factor
ΔΨm: Mitochondrial Membrane Potential

## References

[B1] Akter S. Ahmad S. U. Bhuiyan M. A. Dewan I. Reza R. Morshed N. (2025). Network pharmacology, molecular docking and experimental validation on potential application of diabetic wound healing of Cinnamomum zeylanicum through matrix Metalloproteinases-8 and 9 (MMP-8 and MMP-9). Drug Design, Development Therapy 19, 1753–1782. 10.2147/DDDT.S489113 40093644 PMC11910940

[B2] Akude E. Zherebitskaya E. Chowdhury S. K. Smith D. R. Dobrowsky R. T. Fernyhough P. (2011). Diminished superoxide generation is associated with respiratory chain dysfunction and changes in the mitochondrial proteome of sensory neurons from diabetic rats. Diabetes 60, 288–297. 10.2337/db10-0818 20876714 PMC3012184

[B3] Ali R. E. Rattan S. I. (2006). Curcumin's biphasic hormetic response on proteasome activity and heat-shock protein synthesis in human keratinocytes. Ann. N. Y. Acad. Sci. 1067, 394–399. 10.1196/annals.1354.056 16804017

[B4] Arcos M. Goodla L. Kim H. Desai S. P. Liu R. Yin K. (2025). PINK1-deficiency facilitates mitochondrial iron accumulation and Colon tumorigenesis. Autophagy 21, 737–753. 10.1080/15548627.2024.2425594 39512202 PMC12506718

[B5] Barja G. Herrero A. (2000). Oxidative damage to mitochondrial DNA is inversely related to maximum life span in the heart and brain of mammals. FASEB Journal Official Publication Fed. Am. Soc. Exp. Biol. 14, 312–318. 10.1096/fasebj.14.2.312 10657987

[B6] Bauernfeind F. Bartok E. Rieger A. Franchi L. Núñez G. Hornung V. (2011). Cutting edge: reactive oxygen species inhibitors block priming, but not activation, of the NLRP3 inflammasome. J. Immunology Baltim. Md 1950 187, 613–617. 10.4049/jimmunol.1100613 21677136 PMC3131480

[B7] Bayet-Robert M. Morvan D. (2013). Metabolomics reveals metabolic targets and biphasic responses in breast cancer cells treated by curcumin alone and in association with docetaxel. PloS One 8, e57971. 10.1371/journal.pone.0057971 23472124 PMC3589461

[B8] Beegum F. P V. A. George K. T. K P D. Begum F. Krishnadas N. (2022). Sirtuins as therapeutic targets for improving delayed wound healing in diabetes. J. Drug Targeting 30, 911–926. 10.1080/1061186X.2022.2085729 35787722

[B9] Bharath L. P. Agrawal M. McCambridge G. Nicholas D. A. Hasturk H. Liu J. (2020). Metformin enhances autophagy and normalizes mitochondrial function to alleviate aging-associated inflammation. Cell Metab. 32, 44–55.e6. 10.1016/j.cmet.2020.04.015 32402267 PMC7217133

[B10] Bian D. Wu Y. Song G. Azizi R. Zamani A. (2022). The application of mesenchymal stromal cells (MSCs) and their derivative exosome in skin wound healing: a comprehensive review. Stem Cell Research and Therapy 13, 24. 10.1186/s13287-021-02697-9 35073970 PMC8785459

[B11] Billingham L. K. Stoolman J. S. Vasan K. Rodriguez A. E. Poor T. A. Szibor M. (2022). Mitochondrial electron transport chain is necessary for NLRP3 inflammasome activation. Nat. Immunology 23, 692–704. 10.1038/s41590-022-01185-3 35484407 PMC9098388

[B12] Birk A. V. Liu S. Soong Y. Mills W. Singh P. Warren J. D. (2013). The mitochondrial-targeted compound SS-31 re-energizes ischemic mitochondria by interacting with cardiolipin. J. Am. Soc. Nephrol. JASN 24, 1250–1261. 10.1681/ASN.2012121216 23813215 PMC3736700

[B13] Bond S. T. Kim J. Calkin A. C. Drew B. G. (2019). The antioxidant moiety of MitoQ imparts minimal metabolic effects in adipose tissue of high fat fed mice. Front. Physiology 10, 543. 10.3389/fphys.2019.00543 31139092 PMC6517842

[B14] Bragg S. Marrison S. T. Haley S. (2024). Diabetic peripheral neuropathy: prevention and treatment. Am. Family Physician 109, 226–232. 38574212

[B15] Burnstock G. Knight G. E. Greig A. V. (2012). Purinergic signaling in healthy and diseased skin. J. Investigative Dermatology 132, 526–546. 10.1038/jid.2011.344 22158558

[B16] Cano Sanchez M. Lancel S. Boulanger E. Neviere R. (2018). Targeting oxidative stress and mitochondrial dysfunction in the treatment of impaired wound healing: a systematic review. Antioxidants Basel, Switz. 7 (8), 98. 10.3390/antiox7080098 30042332 PMC6115926

[B17] Cao M. Jiang J. Du Y. Yan P. (2012). Mitochondria-targeted antioxidant attenuates high glucose-induced P38 MAPK pathway activation in human neuroblastoma cells. Mol. Medicine Reports 5, 929–934. 10.3892/mmr.2012.746 22245807 PMC3493100

[B18] Chan D. C. (2020). Mitochondrial dynamics and its involvement in disease. Annu. Review Pathology 15, 235–259. 10.1146/annurev-pathmechdis-012419-032711 31585519

[B19] Chavez J. D. Tang X. Campbell M. D. Reyes G. Kramer P. A. Stuppard R. (2020). Mitochondrial protein interaction landscape of SS-31. Proc. Natl. Acad. Sci. U. S. A. 117, 15363–15373. 10.1073/pnas.2002250117 32554501 PMC7334473

[B20] Chen W. Zhao H. Li Y. (2023a). Mitochondrial dynamics in health and disease: mechanisms and potential targets. Signal Transduction Targeted Therapy 8, 333. 10.1038/s41392-023-01547-9 37669960 PMC10480456

[B21] Chen Y. Chen X. Zhou Q. (2023b). Different effects of a perioperative single dose of dexamethasone on wound healing in mice with or without sepsis. Front. Surgery 10, 927168. 10.3389/fsurg.2023.927168 37114154 PMC10126451

[B22] Chen Z. Wang L. Guo C. Qiu M. Cheng L. Chen K. (2023c). Vascularized polypeptide hydrogel modulates macrophage polarization for wound healing. Acta Biomater. 155, 218–234. 10.1016/j.actbio.2022.11.002 36396041

[B23] Chen P. Vilorio N. C. Dhatariya K. Jeffcoate W. Lobmann R. McIntosh C. (2024). Guidelines on interventions to enhance healing of foot ulcers in people with diabetes (IWGDF 2023 update). Diabetes/metabolism Research Reviews 40, e3644. 10.1002/dmrr.3644 37232034

[B24] Chu M. Wang H. Bian L. Huang J. Wu D. Zhang R. (2022). Nebulization therapy with umbilical cord Mesenchymal stem cell-derived exosomes for COVID-19 Pneumonia. Stem Cell Reviews Reports 18, 2152–2163. 10.1007/s12015-022-10398-w 35665467 PMC9166932

[B25] Ciccone L. Piragine E. Brogi S. Camodeca C. Fucci R. Calderone V. (2022). Resveratrol-like compounds as SIRT1 activators. Int. Journal Molecular Sciences 23, 15105. 10.3390/ijms232315105 36499460 PMC9738298

[B26] Corbi G. Nobile V. Conti V. Cannavo A. Sorrenti V. Medoro A. (2023). Equol and resveratrol improve bone turnover biomarkers in postmenopausal women: a clinical trial. Int. Journal Molecular Sciences 24, 12063. 10.3390/ijms241512063 37569440 PMC10419295

[B27] Cui X. Huang C. Huang Y. Zhang Y. Wu J. Wang G. (2024). Amplification of metalloregulatory proteins in macrophages by bioactive ZnMn@SF hydrogels for spinal cord Injury repair. ACS Nano 18, 33614–33628. 10.1021/acsnano.4c12236 39579147

[B28] Dai S. Yao L. Liu L. Cui J. Su Z. Zhao A. (2024). Carbon dots-supported Zn single atom nanozymes for the catalytic therapy of diabetic wounds. Acta Biomater. 186, 454–469. 10.1016/j.actbio.2024.07.045 39098446

[B29] Deng Q. S. Gao Y. Rui B. Y. Li X. R. Liu P. L. Han Z. Y. (2023). Double-network hydrogel enhanced by SS31-loaded mesoporous polydopamine nanoparticles: symphonic collaboration of near-infrared photothermal antibacterial effect and mitochondrial maintenance for full-thickness wound healing in diabetes mellitus. Bioact. Materials 27, 409–428. 10.1016/j.bioactmat.2023.04.004 37152712 PMC10160601

[B30] Ding R. Li H. Liu Y. Ou W. Zhang X. Chai H. (2022). Activating cGAS-STING axis contributes to neuroinflammation in CVST mouse model and induces inflammasome activation and microglia pyroptosis. J. Neuroinflammation 19, 137. 10.1186/s12974-022-02511-0 35689216 PMC9188164

[B31] Ding Z. Yang C. Zhai X. Xia Y. Liu J. Yu M. (2025). Polyethylene glycol loxenatide accelerates diabetic wound healing by downregulating systemic inflammation and improving endothelial progenitor cell functions. Int. Journal Molecular Sciences 26, 2367. 10.3390/ijms26052367 40076985 PMC11901084

[B32] Djordjević V. V. Kostić J. Krivokapić Ž. Krtinić D. Ranković M. Petković M. (2022). Decreased activity of Erythrocyte catalase and glutathione peroxidase in patients with schizophrenia. Med. Kaunas. Lith. 58, 1491. 10.3390/medicina58101491 36295651 PMC9609318

[B33] Dong M. W. Li M. Chen J. Fu T. T. Lin K. Z. Ye G. H. (2016). Activation of α7nAChR promotes diabetic wound healing by suppressing AGE-Induced TNF-α production. Inflammation 39, 687–699. 10.1007/s10753-015-0295-x 26650489

[B34] Elajaili H. Lyttle B. D. Lewis C. V. Bardill J. R. Dee N. Seal S. (2025). Increased ROS and persistent pro-inflammatory responses in a diabetic wound healing model (Db/db): implications for delayed wound healing. Int. Journal Molecular Sciences 26, 4884. 10.3390/ijms26104884 40430024 PMC12112478

[B35] Erol Doğan Ö. Karaca Çelik K. E. Baş M. Alan E. H. Çağın Y. F. (2024). Effects of mediterranean Diet, Curcumin, and resveratrol on mild-to-moderate active ulcerative colitis: a multicenter randomized clinical trial. Nutrients 16, 1504. 10.3390/nu16101504 38794742 PMC11123867

[B36] Farabi B. Roster K. Hirani R. Tepper K. Atak M. F. Safai B. (2024). The efficacy of stem cells in wound healing: a systematic review. Int. Journal Molecular Sciences 25, 3006. 10.3390/ijms25053006 38474251 PMC10931571

[B37] Feng S. W. Chang P. C. Chen H. Y. Hueng D. Y. Li Y. F. Huang S. M. (2022). Exploring the mechanism of adjuvant treatment of glioblastoma using temozolomide and metformin. Int. Journal Molecular Sciences 23, 8171. 10.3390/ijms23158171 35897747 PMC9330793

[B38] Frykberg R. G. (2021). Topical wound oxygen therapy in the treatment of chronic diabetic foot ulcers. Med. Kaunas. Lith. 57, 917. 10.3390/medicina57090917 34577840 PMC8467973

[B39] Fu Y. J. Shi Y. F. Wang L. Y. Zhao Y. F. Wang R. K. Li K. (2023). All-Natural immunomodulatory bioadhesive hydrogel promotes angiogenesis and diabetic wound healing by regulating macrophage heterogeneity. Adv. Science Weinheim, Baden-Wurttemberg, Ger. 10, e2206771. 10.1002/advs.202206771 36862027 PMC10161050

[B40] Fuhrmann D. C. Wittig I. Brüne B. (2019). TMEM126B deficiency reduces mitochondrial SDH oxidation by LPS, attenuating HIF-1α stabilization and IL-1β expression. Redox Biology 20, 204–216. 10.1016/j.redox.2018.10.007 30368040 PMC6202876

[B41] Geng K. Ma X. Jiang Z. Huang W. Gao C. Pu Y. (2021). Innate immunity in diabetic wound healing: focus on the mastermind hidden in chronic inflammatory. Front. Pharmacology 12, 653940. 10.3389/fphar.2021.653940 33967796 PMC8097165

[B42] Geng K. Ma X. Jiang Z. Huang W. Gu J. Wang P. (2023). High glucose-induced STING activation inhibits diabetic wound healing through promoting M1 polarization of macrophages. Cell Death Discovery 9, 136. 10.1038/s41420-023-01425-x 37100799 PMC10133226

[B43] Geng X. Wang Y. Li H. Song L. Luo C. Gu X. (2024). Total iridoid glycoside extract of Lamiophlomis rotata (Benth) Kudo accelerates diabetic wound healing by the NRF2/COX2 axis. Chin. Medicine 19, 53. 10.1186/s13020-024-00921-1 38519940 PMC10960394

[B44] Gerő D. Torregrossa R. Perry A. Waters A. Le-Trionnaire S. Whatmore J. L. (2016). The novel mitochondria-targeted hydrogen sulfide (H(2)S) donors AP123 and AP39 protect against hyperglycemic injury in microvascular endothelial cells *in vitro* . Pharmacol. Research 113, 186–198. 10.1016/j.phrs.2016.08.019 27565382 PMC5113977

[B45] Gong Y. Wang P. Cao R. Wu J. Ji H. Wang M. (2023). Exudate absorbing and antimicrobial Hydrogel integrated with multifunctional curcumin-loaded magnesium polyphenol network for facilitating burn wound healing. ACS Nano 17, 22355–22370. 10.1021/acsnano.3c04556 37930078

[B46] Guan S. Zhao L. Peng R. (2022). Mitochondrial respiratory chain supercomplexes: from structure to function. Int. Journal Molecular Sciences 23, 13880. 10.3390/ijms232213880 36430359 PMC9696846

[B47] Guo S. Dipietro L. A. (2010). Factors affecting wound healing. J. Dental Research 89, 219–229. 10.1177/0022034509359125 20139336 PMC2903966

[B48] Hayakawa M. Hattori K. Sugiyama S. Ozawa T. (1992). Age-associated oxygen damage and mutations in mitochondrial DNA in human hearts. Biochem. Biophysical Research Communications 189, 979–985. 10.1016/0006-291x(92)92300-m 1472070

[B49] He X. Wang L. Chen X. F. Liang Q. Wang W. Q. Lin A. Q. (2019). Metformin improved oxidized low-density lipoprotein-impaired mitochondrial function and increased glucose uptake involving Akt-AS160 pathway in raw264.7 macrophages. Chin. Medical Journal 132, 1713–1722. 10.1097/CM9.0000000000000333 31268904 PMC6759109

[B50] He F. Huang Y. Song Z. Zhou H. J. Zhang H. Perry R. J. (2021). Mitophagy-mediated adipose inflammation contributes to type 2 diabetes with hepatic insulin resistance. J. Experimental Medicine 218, e20201416. 10.1084/jem.20201416 33315085 PMC7927432

[B51] He S. Li Z. Wang L. Yao N. Wen H. Yuan H. (2024). A nanoenzyme-modified hydrogel targets macrophage reprogramming-angiogenesis crosstalk to boost diabetic wound repair. Bioact. Materials 35, 17–30. 10.1016/j.bioactmat.2024.01.005 38304915 PMC10831190

[B52] He J. Chen J. Liu T. Qin F. Wei W. (2025). Research progress of multifunctional hydrogels in promoting wound healing of diabetes. Int. Journal Nanomedicine 20, 7549–7578. 10.2147/IJN.S519100 40546804 PMC12180465

[B53] Hu N. Wang C. Dai X. Zhou M. Gong L. Yu L. (2020). Phillygenin inhibits LPS-induced activation and inflammation of LX2 cells by TLR4/MyD88/NF-κB signaling pathway. J. Ethnopharmacology 248, 112361. 10.1016/j.jep.2019.112361 31683033

[B54] Hu H. Sheng Q. Yang F. Wu X. Zhang Y. Wu S. (2025). Enhanced skin wound healing through chemically modified messenger RNA encoding Epidermal Growth Factor (EGF). Int. Wound Journal 22, e70143. 10.1111/iwj.70143 40320617 PMC12050261

[B55] Huang J. Yang R. Jiao J. Li Z. Wang P. Liu Y. (2023). A click chemistry-mediated all-peptide cell printing hydrogel platform for diabetic wound healing. Nat. Communications 14, 7856. 10.1038/s41467-023-43364-2 38030636 PMC10687272

[B56] Huang X. Lin Z. Ruan M. Huang P. Ding H. Pan H. (2025). Light up the mitochondria: smart tungstate-oligosaccharide nanoplatform orchestrates mitochondrial transfer from M2 macrophages to restore endothelial function for adaptive diabetic wound regeneration. Mater. Today Bio 34, 102196. 10.1016/j.mtbio.2025.102196 40893353 PMC12391290

[B57] Jang Y. Lee A. Y. Jeong S. H. Park K. H. Paik M. K. Cho N. J. (2015). Chlorpyrifos induces NLRP3 inflammasome and pyroptosis/apoptosis *via* mitochondrial oxidative stress in human keratinocyte HaCaT cells. Toxicology 338, 37–46. 10.1016/j.tox.2015.09.006 26435000

[B58] Jha J. C. Dai A. Garzarella J. Charlton A. Urner S. Østergaard J. A. (2022). Independent of renox, NOX5 promotes renal inflammation and fibrosis in diabetes by activating ROS-Sensitive pathways. Diabetes 71, 1282–1298. 10.2337/db21-1079 35275988

[B59] Jian K. Yang C. Li T. Wu X. Shen J. Wei J. (2022). PDGF-BB-derived supramolecular hydrogel for promoting skin wound healing. J. Nanobiotechnology 20, 201. 10.1186/s12951-022-01390-0 35473604 PMC9044828

[B60] Jomova K. Alomar S. Y. Alwasel S. H. Nepovimova E. Kuca K. Valko M. (2024). Several lines of antioxidant defense against oxidative stress: antioxidant enzymes, nanomaterials with multiple enzyme-mimicking activities, and low-molecular-weight antioxidants. Archives Toxicology 98, 1323–1367. 10.1007/s00204-024-03696-4 38483584 PMC11303474

[B61] Kim J. Y. Lee S. H. Bae I. H. Shin D. W. Min D. Ham M. (2018a). Pyruvate protects against cellular senescence through the control of mitochondrial and lysosomal function in dermal fibroblasts. J. Investigative Dermatology 138, 2522–2530. 10.1016/j.jid.2018.05.033 29959907

[B62] Kim Y. M. Youn S. W. Sudhahar V. Das A. Chandhri R. Cuervo Grajal H. (2018b). Redox regulation of mitochondrial fission protein Drp1 by protein disulfide isomerase limits endothelial senescence. Cell Reports 23, 3565–3578. 10.1016/j.celrep.2018.05.054 29924999 PMC6324937

[B63] Koo J. H. Jang H. Y. Lee Y. Moon Y. J. Bae E. J. Yun S. K. (2019). Myeloid cell-specific sirtuin 6 deficiency delays wound healing in mice by modulating inflammation and macrophage phenotypes. Exp. and Molecular Medicine 51, 1–10. 10.1038/s12276-019-0248-9 31028245 PMC6486573

[B64] Koo S. Sohn H. S. Kim T. H. Yang S. Jang S. Y. Ye S. (2023). Ceria-vesicle nanohybrid therapeutic for modulation of innate and adaptive immunity in a collagen-induced arthritis model. Nat. Nanotechnology 18, 1502–1514. 10.1038/s41565-023-01523-y 37884660

[B65] Lazarou M. Sliter D. A. Kane L. A. Sarraf S. A. Wang C. Burman J. L. (2015). The ubiquitin kinase PINK1 recruits autophagy receptors to induce mitophagy. Nature 524, 309–314. 10.1038/nature14893 26266977 PMC5018156

[B66] Li X. Liu R. Su X. Pan Y. Han X. Shao C. (2019). Harnessing tumor-associated macrophages as aids for cancer immunotherapy. Mol. Cancer 18, 177. 10.1186/s12943-019-1102-3 31805946 PMC6894344

[B67] Li Q. Liao J. Chen W. Zhang K. Li H. Ma F. (2022a). NAC alleviative ferroptosis in diabetic nephropathy *via* maintaining mitochondrial redox homeostasis through activating SIRT3-SOD2/Gpx4 pathway. Free Radical Biology and Medicine 187, 158–170. 10.1016/j.freeradbiomed.2022.05.024 35660452

[B68] Li A. Gao M. Liu B. Qin Y. Chen L. Liu H. (2022b). Mitochondrial autophagy: molecular mechanisms and implications for cardiovascular disease. Cell Death and Disease 13, 444. 10.1038/s41419-022-04906-6 35534453 PMC9085840

[B69] Li J. Y. Sun X. A. Wang X. Yang N. H. Xie H. Y. Guo H. J. (2024a). PGAM5 exacerbates acute renal injury by initiating mitochondria-dependent apoptosis by facilitating mitochondrial cytochrome c release. Acta Pharmacologica Sin. 45, 125–136. 10.1038/s41401-023-01151-1 37684381 PMC10770374

[B70] Li F. Mao Z. Du Y. Cui Y. Yang S. Huang K. (2024b). Mesoporous MOFs with ROS scavenging capacity for the alleviation of inflammation through inhibiting stimulator of interferon genes to promote diabetic wound healing. J. Nanobiotechnology 22, 246. 10.1186/s12951-024-02423-6 38735970 PMC11089722

[B71] Liang C. Padavannil A. Zhang S. Beh S. Robinson D. R. L. Meisterknecht J. (2025). Formation of I(2)+III(2) supercomplex rescues respiratory chain defects. Cell Metab. 37, 441–459.e11. 10.1016/j.cmet.2024.11.011 39788125 PMC11892702

[B72] Lightner A. L. Sengupta V. Qian S. Ransom J. T. Suzuki S. Park D. J. (2023). Bone marrow mesenchymal stem cell-derived extracellular vesicle infusion for the treatment of respiratory failure from COVID-19: a randomized, placebo-controlled dosing clinical trial. Chest 164, 1444–1453. 10.1016/j.chest.2023.06.024 37356708 PMC10289818

[B73] Lin Q. Li S. Jiang N. Shao X. Zhang M. Jin H. (2019). PINK1-parkin pathway of mitophagy protects against contrast-induced acute kidney injury *via* decreasing mitochondrial ROS and NLRP3 inflammasome activation. Redox Biology 26, 101254. 10.1016/j.redox.2019.101254 31229841 PMC6597739

[B74] Liu D. Jin F. Shu G. Xu X. Qi J. Kang X. (2019). Enhanced efficiency of mitochondria-targeted peptide SS-31 for acute kidney injury by pH-responsive and AKI-kidney targeted nanopolyplexes. Biomaterials 211, 57–67. 10.1016/j.biomaterials.2019.04.034 31085359

[B75] Liu C. Han Y. Gu X. Li M. Du Y. Feng N. (2021). Paeonol promotes Opa1-mediated mitochondrial fusion *via* activating the CK2α-Stat3 pathway in diabetic cardiomyopathy. Redox Biology 46, 102098. 10.1016/j.redox.2021.102098 34418601 PMC8385203

[B76] Liu Z. Wang M. Wang X. Bu Q. Wang Q. Su W. (2022). XBP1 deficiency promotes hepatocyte pyroptosis by impairing mitophagy to activate mtDNA-cGAS-STING signaling in macrophages during acute liver injury. Redox Biology 52, 102305. 10.1016/j.redox.2022.102305 35367811 PMC8971356

[B77] Liu C. Zhou X. Ju H. Zhang Y. (2023). Inhibition of pyruvate carboxylase reverses metformin resistance by activating AMPK in pancreatic cancer. Life Sciences 327, 121817. 10.1016/j.lfs.2023.121817 37270169

[B78] Liu H. Zhao S. Wang H. He X. Gao S. Su M. (2025). From inflammation to healing: the crucial role of GPR91 activation and SDH inhibition in chronic diabetic wound recovery. Stem Cell Research and Therapy 16, 399. 10.1186/s13287-025-04480-6 40702577 PMC12288363

[B79] Louiselle A. E. Niemiec S. M. Zgheib C. Liechty K. W. (2021). Macrophage polarization and diabetic wound healing. Transl. Research The Journal Laboratory Clinical Medicine 236, 109–116. 10.1016/j.trsl.2021.05.006 34089902

[B80] Lu W. Li X. Wang Z. Zhao C. Li Q. Zhang L. (2024). Mesenchymal stem cell-derived extracellular vesicles accelerate diabetic wound healing by inhibiting NET-Induced ferroptosis of endothelial cells. Int. Journal Biological Sciences 20, 3515–3529. 10.7150/ijbs.97150 38993565 PMC11234223

[B81] Luo Y. Vivaldi M. E. Choudhary V. Bollag W. B. (2023). Phosphatidylglycerol to treat chronic skin wounds in diabetes. Pharmaceutics 15 (5), 1497. 10.3390/pharmaceutics15051497 37242739 PMC10222993

[B82] Mahmoud N. N. Hamad S. Shraim S. (2024). Inflammation-modulating biomedical interventions for diabetic wound healing: an overview of preclinical and clinical studies. ACS Omega 9, 44860–44875. 10.1021/acsomega.4c02251 39554458 PMC11561615

[B83] Martin P. Nunan R. (2015). Cellular and molecular mechanisms of repair in acute and chronic wound healing. Br. Journal Dermatology 173, 370–378. 10.1111/bjd.13954 26175283 PMC4671308

[B84] McMinimy R. Manford A. G. Gee C. L. Chandrasekhar S. Mousa G. A. Chuang J. (2024). Reactive oxygen species control protein degradation at the mitochondrial import gate. Mol. Cell 84, 4612–4628.e13. 10.1016/j.molcel.2024.11.004 39642856 PMC11649020

[B85] Mendoza A. Patel P. Robichaux D. Ramirez D. Karch J. (2024). Inhibition of the mPTP and lipid peroxidation is additively protective against I/R injury. Circulation Research 134, 1292–1305. 10.1161/CIRCRESAHA.123.323882 38618716 PMC11081482

[B86] Monteiro L. B. Prodonoff J. S. Favero de Aguiar C. Correa-da-Silva F. Castoldi A. Bakker N. v. T. (2022). Leptin signaling suppression in macrophages improves immunometabolic outcomes in obesity. Diabetes 71, 1546–1561. 10.2337/db21-0842 35377454

[B87] Muñoz-Planillo R. Kuffa P. Martínez-Colón G. Smith B. L. Rajendiran T. M. Núñez G. (2013). K^+^ efflux is the common trigger of NLRP3 inflammasome activation by bacterial toxins and particulate matter. Immunity 38, 1142–1153. 10.1016/j.immuni.2013.05.016 23809161 PMC3730833

[B88] Naziroğlu M. (2007). New molecular mechanisms on the activation of TRPM2 channels by oxidative stress and ADP-Ribose. Neurochem. Research 32, 1990–2001. 10.1007/s11064-007-9386-x 17562166

[B89] Narendra D. P. Youle R. J. (2024). The role of PINK1-Parkin in mitochondrial quality control. Nat. Cell Biology 26, 1639–1651. 10.1038/s41556-024-01513-9 39358449

[B90] Nedosugova L. V. Markina Y. V. Bochkareva L. A. Kuzina I. A. Petunina N. A. Yudina I. Y. (2022). Inflammatory mechanisms of diabetes and its vascular complications. Biomedicines 10, 1168. 10.3390/biomedicines10051168 35625904 PMC9138517

[B91] Ou M. Y. Tan P. C. Xie Y. Liu K. Gao Y. M. Yang X. S. (2022). Dedifferentiated schwann cell-derived TGF-β3 is essential for the neural system to promote wound healing. Theranostics 12, 5470–5487. 10.7150/thno.72317 35910794 PMC9330527

[B92] Öz A. Çelik Ö. (2016). Curcumin inhibits oxidative stress-induced TRPM2 channel activation, calcium ion entry and apoptosis values in SH-SY5Y neuroblastoma cells: involvement of transfection procedure. Mol. Membrane Biology 33, 76–88. 10.1080/09687688.2017.1318224 28569571

[B93] Peña O. A. Martin P. (2024). Cellular and molecular mechanisms of skin wound healing. Nat. Reviews Mol. Cell Biology 25, 599–616. 10.1038/s41580-024-00715-1 38528155

[B94] Peng Z. Gillissen B. Richter A. Sinnberg T. Schlaak M. S. Eberle J. (2023). Enhanced apoptosis and loss of cell viability in melanoma cells by combined inhibition of ERK and Mcl-1 is related to loss of mitochondrial membrane potential, caspase activation and upregulation of proapoptotic Bcl-2 proteins. Int. Journal Molecular Sciences 24, 4961. 10.3390/ijms24054961 36902392 PMC10002974

[B95] Piper J. T. Singhal S. S. Salameh M. S. Torman R. T. Awasthi Y. C. Awasthi S. (1998). Mechanisms of anticarcinogenic properties of curcumin: the effect of curcumin on glutathione linked detoxification enzymes in rat liver. International Journal Biochemistry and Cell Biology 30, 445–456. 10.1016/s1357-2725(98)00015-6 9675878

[B96] Purvis G. S. D. Collino M. van Dam A. D. Einaudi G. Ng Y. Shanmuganathan M. (2024). OxPhos in adipose tissue macrophages regulated by BTK enhances their M2-like phenotype and confers a systemic immunometabolic benefit in obesity. Diabetes. db220275, 10.2337/db22-0275 38193882

[B97] Qi X. Liu C. Si J. Yin B. Huang J. Wang X. (2024). A bioenergetically-active ploy (glycerol sebacate)-based multiblock hydrogel improved diabetic wound healing through revitalizing mitochondrial metabolism. Cell Proliferation 57, e13613. 10.1111/cpr.13613 38351579 PMC11216945

[B98] Qian B. Li J. Guo K. Guo N. Zhong A. Yang J. (2021). Antioxidant biocompatible composite collagen dressing for diabetic wound healing in rat model. Regen. Biomaterials 8, rbab003. 10.1093/rb/rbab003 33738117 PMC7955720

[B99] Qin D. Hu W. Guo Y. Cheng R. Hao F. Zhao B. (2025). Baicalein based nano-delivery system restores mitochondrial homeostasis through PPAR signaling pathway to promote wound healing in diabetes. J. Nanobiotechnology 23, 360. 10.1186/s12951-025-03427-6 40383752 PMC12087252

[B100] Quintana-Cabrera R. Scorrano L. (2023). Determinants and outcomes of mitochondrial dynamics. Mol. Cell 83, 857–876. 10.1016/j.molcel.2023.02.012 36889315

[B101] Renò F. Pagano C. A. Bignotto M. Sabbatini M. (2025). Neutrophil heterogeneity in wound healing. Biomedicines 13 (3), 694. 10.3390/biomedicines13030694 40149670 PMC11940162

[B102] Sá-Pessoa J. López-Montesino S. Przybyszewska K. Rodríguez-Escudero I. Marshall H. Ova A. (2023). A trans-kingdom T6SS effector induces the fragmentation of the mitochondrial network and activates innate immune receptor NLRX1 to promote infection. Nat. Communications 14, 871. 10.1038/s41467-023-36629-3 36797302 PMC9935632

[B103] Scharstuhl A. Mutsaers H. A. Pennings S. W. Szarek W. A. Russel F. G. M. Wagener F. A. D. T. G. (2009). Curcumin-induced fibroblast apoptosis and *in vitro* wound contraction are regulated by antioxidants and heme oxygenase: implications for scar formation. J. Cellular Molecular Medicine 13, 712–725. 10.1111/j.1582-4934.2008.00339.x 18410527 PMC3822878

[B104] Senneville É. Albalawi Z. van Asten S. A. Abbas Z. G. Allison G. Aragón-Sánchez J. (2024). IWGDF/IDSA guidelines on the diagnosis and treatment of diabetes-related foot infections (IWGDF/IDSA 2023). Diabetes/metabolism Research Reviews 40, e3687. 10.1002/dmrr.3687 37779323

[B105] Shao Y. Zhou X. Zhou S. Long J. Jin L. Shi X. (2025). Injectable DMM/GelMA hydrogel for diabetic wound healing *via* regulating mitochondrial metabolism and macrophage repolarization. Colloids Surfaces B, Biointerfaces 248, 114488. 10.1016/j.colsurfb.2024.114488 39765076

[B106] Sidarala V. Zhu J. Levi-D'Ancona E. Pearson G. L. Reck E. C. Walker E. M. (2022). Mitofusin 1 and 2 regulation of mitochondrial DNA content is a critical determinant of glucose homeostasis. Nat. Communications 13, 2340. 10.1038/s41467-022-29945-7 PMC9055072 35487893

[B107] Sideek S. A. El-Nassan H. B. Fares A. R. ElMeshad A. N. Elkasabgy N. A. (2022). Different curcumin-loaded delivery systems for wound healing applications: a comprehensive review. Pharmaceutics 15, 38. 10.3390/pharmaceutics15010038 36678665 PMC9862251

[B108] Singh P. Gollapalli K. Mangiola S. Schranner D. Yusuf M. A. Chamoli M. (2023). Taurine deficiency as a driver of aging. Sci. (New York, NY) 380, eabn9257. 10.1126/science.abn9257 37289866 PMC10630957

[B109] Sulkshane P. Ram J. Thakur A. Reis N. Kleifeld O. Glickman M. H. (2021). Ubiquitination and receptor-mediated mitophagy converge to eliminate oxidation-damaged mitochondria during hypoxia. Redox Biology 45, 102047. 10.1016/j.redox.2021.102047 34175667 PMC8254004

[B110] Sun J. Liu X. Shen C. Zhang W. Niu Y. (2021). Adiponectin receptor agonist AdipoRon blocks skin inflamm-ageing by regulating mitochondrial dynamics. Cell Proliferation 54, e13155. 10.1111/cpr.13155 34725875 PMC8666283

[B111] Sun K. X. Chen Y. Y. Li Z. Zheng S. J. Wan W. J. Ji Y. (2023). Genipin relieves diabetic retinopathy by down-regulation of advanced glycation end products *via* the mitochondrial metabolism related signaling pathway. World Journal Diabetes 14, 1349–1368. 10.4239/wjd.v14.i9.1349 37771331 PMC10523227

[B112] Sweetwyne M. T. Pippin J. W. Eng D. G. Hudkins K. L. Chiao Y. A. Campbell M. D. (2017). The mitochondrial-targeted peptide, SS-31, improves glomerular architecture in mice of advanced age. Kidney International 91, 1126–1145. 10.1016/j.kint.2016.10.036 28063595 PMC5392164

[B113] Szeto H. H. (2014). First-in-class cardiolipin-protective compound as a therapeutic agent to restore mitochondrial bioenergetics. Br. Journal Pharmacology 171, 2029–2050. 10.1111/bph.12461 24117165 PMC3976620

[B114] Szeto H. H. Birk A. V. (2014). Serendipity and the discovery of novel compounds that restore mitochondrial plasticity. Clin. Pharmacology Therapeutics 96, 672–683. 10.1038/clpt.2014.174 25188726 PMC4267688

[B115] Tang S. Huang M. Wang R. Dong N. Wu R. Chi Z. (2024). Drp1-dependent mitochondrial fragmentation mediates photoreceptor abnormalities in type 1 diabetic retina. Exp. Eye Research 242, 109860. 10.1016/j.exer.2024.109860 38467174

[B116] Tannahill G. M. Curtis A. M. Adamik J. Palsson-McDermott E. M. McGettrick A. F. Goel G. (2013). Succinate is an inflammatory signal that induces IL-1β through HIF-1α. Nature 496, 238–242. 10.1038/nature11986 23535595 PMC4031686

[B117] Thomas C. Mackey M. M. Diaz A. A. Cox D. P. (2009). Hydroxyl radical is produced *via* the fenton reaction in submitochondrial particles under oxidative stress: implications for diseases associated with iron accumulation. Redox Report Communications Free Radical Research 14, 102–108. 10.1179/135100009X392566 19490751

[B118] Tian S. Mei J. Zhang L. Wang S. Yuan Y. Li J. (2024). Multifunctional hydrogel microneedle patches modulating oxi-inflamm-aging for diabetic wound healing. Small Weinheim der Bergstrasse, Ger. 20, e2407340. 10.1002/smll.202407340 39360460

[B119] Trambas I. A. Bowen L. Thallas-Bonke V. Snelson M. Sourris K. C. Laskowski A. (2025). Proximal tubular deletion of superoxide dismutase-2 reveals disparate effects on kidney function in diabetes. Redox Biology 82, 103601. 10.1016/j.redox.2025.103601 40127616 PMC11979990

[B120] Van den Bossche J. Baardman J. de Winther M. P. (2015). Metabolic characterization of polarized M1 and M2 bone marrow-derived macrophages using real-time extracellular flux analysis. J. Visualized Experiments JoVE. 105, 53424. 10.3791/53424 26649578 PMC4692751

[B121] Vringer E. Heilig R. Riley J. S. Black A. Cloix C. Skalka G. (2024). Mitochondrial outer membrane integrity regulates a ubiquitin-dependent and NF-κB-mediated inflammatory response. EMBO Journal 43, 904–930. 10.1038/s44318-024-00044-1 38337057 PMC10943237

[B122] Wang G. Wang Y. Yang Q. Xu C. Zheng Y. Wang L. (2022a). Metformin prevents methylglyoxal-induced apoptosis by suppressing oxidative stress *in vitro* and *in vivo* . Cell Death and Disease 13, 29. 10.1038/s41419-021-04478-x 35013107 PMC8748764

[B123] Wang L. Wang H. Luo J. Xie T. Mor G. Liao A. (2022b). Decorin promotes decidual M1-like macrophage polarization *via* mitochondrial dysfunction resulting in recurrent pregnancy loss. Theranostics 12, 7216–7236. 10.7150/thno.78467 36438479 PMC9691373

[B124] Wang Y. Chen J. Zheng Y. Jiang J. Wang L. Wu J. (2024). Glucose metabolite methylglyoxal induces vascular endothelial cell pyroptosis *via* NLRP3 inflammasome activation and oxidative stress *in vitro* and *in vivo* . Cell. Molecular Life Sciences CMLS 81, 401. 10.1007/s00018-024-05432-8 39269632 PMC11399538

[B125] Wang Y. Ni T. Zhang Q. Xu Z. Zhu Z. Xie J. (2025a). AhR deficiency exacerbates inflammation in diabetic wounds *via* impaired mitophagy and cGAS-STING-NLRP3 activation: therapeutic potential of hydrogels loaded with FICZ. Mater. Today Bio 34, 102119. 10.1016/j.mtbio.2025.102119 40755898 PMC12318297

[B126] Wang Y. Liao W. Wang Y. Liao J. Chen N. Jia C. (2025b). Human adipose-derived stem cell exosomes reduce mitochondrial DNA common deletion through PINK1/Parkin-mediated mitophagy to improve skin photoaging. Stem Cell Research and Therapy 16, 365. 10.1186/s13287-025-04475-3 40660391 PMC12261646

[B127] Wu H. Wang Y. Li W. Chen H. Du L. Liu D. (2019). Deficiency of mitophagy receptor FUNDC1 impairs mitochondrial quality and aggravates dietary-induced obesity and metabolic syndrome. Autophagy 15, 1882–1898. 10.1080/15548627.2019.1596482 30898010 PMC6844496

[B128] Wu J. S. Huang H. Y. Wang L. L. Chen W. J. Yuan Q. Li X. Q. (2026). Redox-responsive chitosan hydrogel releases mitochondria-targeted fullerenol to reprogram M1/M2 macrophage balance for diabetic wound healing. J. Controlled Release Official Journal Control. Release Soc. 390, 114506. 10.1016/j.jconrel.2025.114506 41371502

[B129] Xian T. Liu Y. Ye Y. Peng B. Huang J. Liang L. (2024). Human salivary histatin 1 regulating IP3R1/GRP75/VDAC1 mediated mitochondrial-associated endoplasmic reticulum membranes (MAMs) inhibits cell senescence for diabetic wound repair. Free Radical Biology and Medicine 225, 164–180. 10.1016/j.freeradbiomed.2024.09.046 39343182

[B130] Xiao L. Xu X. Zhang F. Wang M. Xu Y. Tang D. (2017). The mitochondria-targeted antioxidant MitoQ ameliorated tubular injury mediated by mitophagy in diabetic kidney disease *via* Nrf2/PINK1. Redox Biology 11, 297–311. 10.1016/j.redox.2016.12.022 28033563 PMC5196243

[B131] Xie Z. Paras C. B. Weng H. Punnakitikashem P. Su L. C. Vu K. (2013). Dual growth factor releasing multi-functional nanofibers for wound healing. Acta Biomater. 9, 9351–9359. 10.1016/j.actbio.2013.07.030 23917148 PMC3818500

[B132] Xie P. Young M. W. Bian H. Niknam-Bienia S. Hong S. Mustoe T. A. (2019). Renal dysfunction aggravated impaired cutaneous wound healing in diabetic mice. Wound Repair Regeneration Official Publication Wound Heal. Soc. Eur. Tissue Repair Soc. 27, 49–58. 10.1111/wrr.12682 30362661

[B133] Xu Z. Liu Y. Ma R. Chen J. Qiu J. Du S. (2022a). Thermosensitive hydrogel incorporating prussian blue nanoparticles promotes diabetic wound healing *via* ROS scavenging and mitochondrial function restoration. ACS Applied Materials and Interfaces 14, 14059–14071. 10.1021/acsami.1c24569 35298140

[B134] Xu L. Yan X. Zhao Y. Wang J. Liu B. Yu S. (2022b). Macrophage polarization mediated by mitochondrial dysfunction induces adipose tissue inflammation in obesity. Int. Journal Molecular Sciences 23, 9252. 10.3390/ijms23169252 36012516 PMC9409464

[B135] Xu D. Moru P. Liao K. Song W. Yang P. Zang D. (2024). High glucose-induced senescence contributes to tubular epithelial cell damage in diabetic nephropathy. Exp. Gerontology 197, 112609. 10.1016/j.exger.2024.112609 39395579

[B136] Xu Z. Ni T. Zhang Q. Sun X. Zhao L. Lin J. (2025). Exosomes derived from fibroblasts in DFUs delay wound healing by delivering miR-93-5p to target macrophage ATG16L1. Biochimica biophysica acta Mol. basis Dis. 1871, 167640. 10.1016/j.bbadis.2024.167640 39761761

[B137] Yang X. Zhan P. Wang X. Zhang Q. Zhang Y. Fan H. (2020). Polydopamine-assisted PDGF-BB immobilization on PLGA fibrous substrate enhances wound healing *via* regulating anti-inflammatory and cytokine secretion. PloS One 15, e0239366. 10.1371/journal.pone.0239366 32991599 PMC7523965

[B138] Yu J. Nagasu H. Murakami T. Hoang H. Broderick L. Hoffman H. M. (2014). Inflammasome activation leads to Caspase-1-dependent mitochondrial damage and block of mitophagy. Proc. Natl. Acad. Sci. U. S. A. 111, 15514–15519. 10.1073/pnas.1414859111 25313054 PMC4217429

[B139] Yu W. Xu H. Yuan X. Chen W. Hao Y. Zhao G. (2025). Cinnamaldehyde promotes diabetic wound healing *via* synergetic effects of AGE/RAGE-mediated macrophage polarization affecting fibroblast activation and angiogenesis, and Nrf2-dependent antioxidants. Biochem. Biophysical Research Communications 781, 152519. 10.1016/j.bbrc.2025.152519 40882313

[B140] Zhang Y. Wang S. Chen X. Wang Z. Wang X. Zhou Q. (2022). Liraglutide prevents high glucose induced HUVECs dysfunction *via* inhibition of PINK1/Parkin-dependent mitophagy. Mol. Cellular Endocrinology 545, 111560. 10.1016/j.mce.2022.111560 35032624

[B141] Zhang Y. Zhang Y. Y. Pan Z. W. Li Q. Q. Sun L. H. Li X. (2023). GDF11 promotes wound healing in diabetic mice *via* stimulating HIF-1ɑ-VEGF/SDF-1ɑ-mediated endothelial progenitor cell mobilization and neovascularization. Acta Pharmacologica Sin. 44, 999–1013. 10.1038/s41401-022-01013-2 36347996 PMC10104842

[B142] Zhang W. Yu W. Zhu Y. Gu J. Gu X. (2024). Alda-1 ameliorates oxidative stress-induced cardiomyocyte damage by inhibiting the mitochondrial ROS/TXNIP/NLRP3 pathway. J. Biochemical Molecular Toxicology 38, e70032. 10.1002/jbt.70032 39467157

[B143] Zhang S. Zhao X. Zhang W. Wei X. Chen X. L. Wang X. (2025a). Zn-DHM nanozymes regulate metabolic and immune homeostasis for early diabetic wound therapy. Bioact. Materials 49, 63–84. 10.1016/j.bioactmat.2025.02.041 40124598 PMC11928983

[B144] Zhang C. Xiang J. Wang G. Di T. Chen L. Zhao W. (2025b). Salvianolic acid B improves diabetic skin wound repair through Pink1/Parkin-mediated mitophagy. Archives Physiology Biochemistry 131, 40–51. 10.1080/13813455.2024.2387693 39101795

[B145] Zhang H. Yan Z. Zhu J. Li Z. Chen L. Zheng W. (2025c). Extracellular mitochondrial-derived vesicles affect the progression of diabetic foot ulcer by regulating oxidative stress and mitochondrial dysfunction. Adv. Science Weinheim, Baden-Wurttemberg, Ger. 12, e2407574. 10.1002/advs.202407574 39835574 PMC11904950

[B146] Zhao W. Xu Z. Cao J. Fu Q. Wu Y. Zhang X. (2019). Elamipretide (SS-31) improves mitochondrial dysfunction, synaptic and memory impairment induced by lipopolysaccharide in mice. J. Neuroinflammation 16, 230. 10.1186/s12974-019-1627-9 31747905 PMC6865061

[B147] Zhao M. Wang Y. Li L. Liu S. Wang C. Yuan Y. (2021). Mitochondrial ROS promote mitochondrial dysfunction and inflammation in ischemic acute kidney injury by disrupting TFAM-mediated mtDNA maintenance. Theranostics 11, 1845–1863. 10.7150/thno.50905 33408785 PMC7778599

[B148] Zhao W. Xu D. Hong W. Zhang L. Wu Q. Gao M. (2022). Grossamide attenuates inflammation by balancing macrophage polarization through metabolic reprogramming of macrophages in mice. Int. Immunopharmacology 112, 109190. 10.1016/j.intimp.2022.109190 36116152

[B149] Zhen A. X. Piao M. J. Kang K. A. Fernando P. D. S. M. Herath H. M. U. L. Cho S. J. (2023). “3-Bromo-4,5-dihydroxybenzaldehyde protects keratinocytes from particulate matter 2.5-Induced damages,” Antioxidants. 1307 12. 10.3390/antiox12061307 37372037 PMC10295767

[B150] Zhou R. Tardivel A. Thorens B. Choi I. Tschopp J. (2010). Thioredoxin-interacting protein links oxidative stress to inflammasome activation. Nat. Immunology 11, 136–140. 10.1038/ni.1831 20023662

[B151] Zhou R. Yazdi A. S. Menu P. Tschopp J. (2011). A role for mitochondria in NLRP3 inflammasome activation. Nature 469, 221–225. 10.1038/nature09663 21124315

[B152] Zhou Z. Deng T. Tao M. Lin L. Sun L. Song X. (2023). Snail-inspired AFG/GelMA hydrogel accelerates diabetic wound healing *via* inflammatory cytokines suppression and macrophage polarization. Biomaterials 299, 122141. 10.1016/j.biomaterials.2023.122141 37167893

[B153] Zhu Y. Yang Y. Li F. Fan S. Chen X. Lu Y. (2019). Stimulation of the class-A scavenger receptor induces neutrophil extracellular traps (NETs) by ERK dependent NOX2 and ROMO1 activation. Biochem. Biophysical Research Communications 511, 847–854. 10.1016/j.bbrc.2019.02.142 30850160

